# Cryo-EM structure and polar assembly of the PS2 S-layer of *Corynebacterium glutamicum*

**DOI:** 10.1073/pnas.2426928122

**Published:** 2025-07-29

**Authors:** Adrià Sogues, Mike Sleutel, Julienne Petit, Daniela Megrian, Nicolas Bayan, Anne Marie Wehenkel, Han Remaut

**Affiliations:** ^a^Structural and Molecular Microbiology, Vlaams Instituut voor Biotechnology (VIB) Center for Structural Biology, Brussels 1050, Belgium; ^b^Structural Biology Brussels, Department for Bio-engineering Sciences, Vrije Universiteit Brussel, VUB, Brussels 1050, Belgium; ^c^Institut Pasteur, Université Paris Cité, CNRS UMR 3528, Bacterial Cell Cycle Mechanisms Unit, Paris F-75015, France; ^d^Institut Pasteur, Université Paris Cité, CNRS UMR 3528, Structural Microbiology Unit, Paris F-75015, France; ^e^Bioinformatics Unit, Institut Pasteur de Montevideo, Montevideo 11200, Uruguay; ^f^Université Paris-Saclay, French Alternative Energies and Atomic Energy Commission, CNRS, Institute for Integrative Biology of the Cell, Gif-sur-Yvette 91190, France

**Keywords:** S-layer, Corynebacterium, cell growth, mycomembrane, biotechnology

## Abstract

The polar-growing *Corynebacteriales* have a complex cell envelope architecture characterized by the presence of a specialized outer membrane composed of mycolic acids. In some *Corynebacteriales*, this mycomembrane is further supported by a proteinaceous surface layer or “S-layer,” whose function, structure, and mode of assembly remain largely enigmatic. Here, we isolated ex vivo PS2 S-layers from the industrially important *Corynebacterium glutamicum* and determined its atomic structure by 3D cryo-EM reconstruction. PS2 monomers consist of a six-helix bundle “core,” a three-helix bundle “arm,” and a C-terminal transmembrane (TM) helix. The PS2 core oligomerizes into hexameric units anchored in the mycomembrane by a channel-like coiled-coil of the TM helices. The PS2 arms mediate trimeric lattice contacts, crystallizing the hexameric units into an intricate semipermeable lattice. Using pulse-chase live cell imaging, we show that the PS2 lattice is incorporated at the poles, coincident with the actinobacterial elongasome. Finally, phylogenetic analysis shows a paraphyletic distribution and dispersed chromosomal location of PS2 in *Corynebacteriales* as a result of multiple recombination events and losses. These findings expand our understanding of S-layer biology and enable applications of membrane-supported self-assembling bioengineered materials.

## Significance

*Corynebacterium glutamicum* is a bacterium commonly used in industry for the production of biobased products. Its cells are coated with a surface layer (S-layer) made from the PS2 protein. We show PS2 consists of umbrella-shaped units that anchor in the outer membrane and arrange into a porous crystal-like lattice around the cells, which helps protect them from external insults. To study how PS2 S-layers grow during the expansion and division of cells, we made a PS2 derivative that can be selectively labeled. In this way, we provide insights into a family of bacteria with an unusual cell growth mechanism, and pave the way for future applications in bacterial surface engineering and the use of S-layers as membrane support materials.

Since the first discovery of a Surface layer (S-layer) over 70 y ago (1), researchers have identified hundreds of S-layers across nearly every bacterial taxonomic group, and in the majority of Archaea. S-layers are two-dimensional monolayered crystals typically composed of a single (glyco)protein that self-assembles to cover the entire cell surface. Considered one of the most abundant protein families on earth, S-layers are often regulated by strong promoters and have stable mRNA half-lives, accounting for 10 to 30% of total protein synthesis and representing a significant energy cost for the cell (2). Many S-layer proteins share a bipartite architecture comprising a cell envelope binding domain and a crystallization domain that self-assembles into 2D lattices of defined symmetry (3). Yet, S-layers often lack discernable sequence or structural homology across different taxonomic groups, indicating their multiple independent emergences across the evolutionary tree of life, likely driven by the advantageous traits they confer as continuous semipermeable nonmembranous layers (4). The reported physiological roles and functions of S-layers are remarkably diverse and may be pleiotropic in many cases (2, 5). Research across various organisms suggests S-layers play roles in adhesion (6), cell-shape maintenance (7), virulence (8), function as molecular sieves (9) or as a cell envelope supporting exoskeleton (10, 11). S-layer function is often hard to discern. Testament to this is the fact that for some characterized S-layers functional data is still lacking as their knockouts show little to no phenotypic difference compared to the WT under the chosen lab conditions, suggesting that their function could be specific to exclusive environmental niches (12). Nevertheless, there are cases where significant differences are observed in the absence of the S-layer. One such example is the extensively studied SlpA S-layer in *Clostridioides difficile*. Absence of a functional *slpA* results in reduced growth, decreased toxin production, impaired sporulation and motility, enhanced biofilm formation, and weaker adhesion to human host cells, highlighting the relevance of the S-layer in this pathogen (13, 14).

**Fig. 1.**
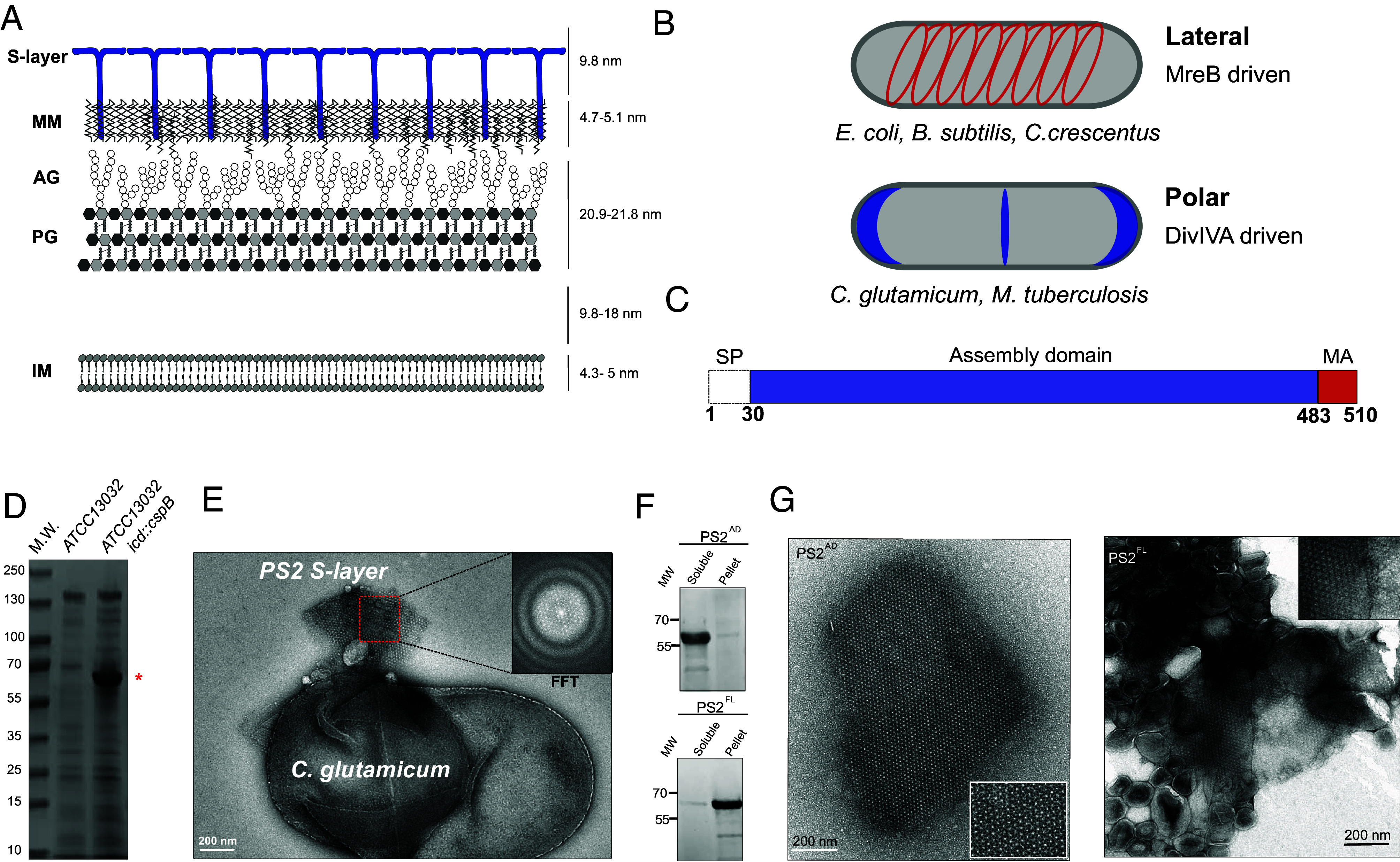
S-layer characterization and domain organization of PS2 S-layer from *C. glutamicum*. (*A*) Schematic representation of the cell surface organization of *C. glutamicum*. IM (Inner membrane); PG (peptidoglycan); AG (arabinogalactan); MM (Mycomembrane). (*B*) Comparison of the growth modes of Actinobacteria (polar) and model organisms *E. coli, C. crescentus,* and *B. subtilis* (lateral). Blue and red represent the localization of DivIVA and MreB respectively which in turn determine PG synthesis. (*C*) Domain organization of PS2. SP (signal peptide); MA (membrane anchor). (*D*) SDS-PAGE of the SDS extracted cell surface protein. The strain expressing PS2 under its native promoter expresses large amounts of PS2 (indicated with *, MW = 55.42 kDa). (*E*) Negative-stained *C.glutamicum* cells expressing PS2 washed with 0.05% SDS. Detached patches of the PS2 S-layers were observed. (*Inset*) Fast Fourier Transform of the ex vivo PS2 S-layer. Estimated unit cell parameters are α = β = 171.8 Å and γ = 60°. (*F*) Western blot analysis (using anti-HisTag antibody) of recombinant PS2 fractionation; assembly domain (AD) and full-length (FL). (*G*) Negative-stained TEM micrograph of in vitro reconstituted recombinant soluble PS2^AD^ (*Left*) and lipid-associated PS2^FL^ (*Right*). (Scale bar, 200 nm.) *Insets* represent zoomed-in to make the lattice pattern more apparent.

Here, we focus on *Corynebacterium glutamicum,* an aerobic, gram-positive soil bacterium that is extensively used in biotechnology and industry for the large-scale biosynthesis of amino acids. Its widespread use is attributed to several advantageous characteristics: biosafety, fast growth to high cell density, genetic stability, absence of autolysis under growth-arrested conditions, low protease activity, and a broad spectrum of carbon source utilization (15). In addition to its growing industrial interest, *C. glutamicum* has emerged as a model organism of the order *Corynebacteriales*, a subgroup of Actinobacteria that includes important human pathogens, such as *Mycobacterium tuberculosis* and *Corynebacterium diphtheriae*. This taxonomic group exhibits specific characteristics distinct from other bacterial model organisms. Two actinobacterial features of interest are the presence of a multilayered cell wall that includes an outer membrane mainly composed of long hydrocarbon chains of mycolic acids either esterified to trehalose or attached to the arabinogalactan polymer, which is, in turn, linked to the peptidoglycan meshwork (Fig. 1*A*) (16); and a unique polar growth mode, characterized by the insertion of new peptidoglycan at the poles, driven by the coiled-coil elongasome scaffold DivIVA (17). This contrasts with model bacilliform organisms such as *Escherichia coli* and *Bacillus subtilis*, which grow laterally by inserting peptidoglycan along the cell wall guided by the actin homologue MreB (18)(Fig. 1*B*).

On top of the mycomembrane, some *Corynebacteriales* species display an S-layer that coats their entire cell surface (Fig. 1*A*). The most studied S-layer protein of this group is the PS2 from *C. glutamicum* (19, 20) whose network has hexagonal (P6) symmetry (20). PS2 is the product of the *cspB* gene (19) and represents the major secreted protein of the cell (21, 22). Early research revealed that PS2 exhibits an abundance of hydrophobic amino acids mainly located in the two terminal regions, identified as an N-terminal signal peptide and a C-terminal cell wall anchoring domain (23) (Fig. 1*C*). As the PS2 S-layer can be stripped from the cell surface by using various detergents it is thought that hydrophobic interactions play a major role in cell wall anchoring. A mutant lacking the 27 C-terminal residues was unable to form an organized cell-bound S-layer and PS2 was mainly released into the medium (23). The current model thus suggests that the C-terminus of PS2 serves as the membrane anchor (MA), tethering the protein to the mycomembrane, which in turn acts as a matrix for 2D crystallization (24) (Fig. 1*C*). The use of membrane-embedded domains for S-layer anchoring is common in Archaea (25) but would represent an uncharacterized mechanism in bacteria. In gram-positive monoderm bacteria, the S-layer is typically bound directly to the peptidoglycan layer through specialized domains, such as the S-layer homology (SLH) domain (26) or cell wall binding (CWB) domains (27). In contrast, in gram-negative diderm bacteria like *Caulobacter crescentus*, the S-layer is anchored to the O-antigen of lipopolysaccharide (28). Therefore, the *Corynebacteriales* order (diderm gram-positive) shows a unique mechanism of S-layer tethering to the cell envelope, likely due to its distinctive cell wall architecture.

In well-studied bacterial S-layers, the crystallinity of the S-layer is essential to the function (8, 10, 11) and growth of the S-layer lattice occurs in spatiotemporal coordination with cell elongation and cell wall synthesis (29–31). Where known, S-layer expansion in bacilliform bacteria occurs by the addition of newly exported subunits onto the edge of the lattice, localized at mid-plane. How S-layer expansion and attachment are adapted to the unique cell envelope features of *Corynebacteriales* is largely unknown.

Here, we present an in-depth analysis of the atomic structure and organization of the PS2 S-layer of *C. glutamicum*. Guided by the lattice structure, we engineered a PS2 variant capable of covalently binding proteins of interest both in vivo and in vitro, making this S-layer a viable target for its use in biomaterials. Using this engineered PS2 and pulse-chase fluorescent labeling, we tracked its assembly in vivo, revealing that this process occurs exclusively at the cell poles. Additionally, an extensive phylogenetic analysis uncovered its scattered distribution within *Corynebacteriales*, along with various genomic contexts, suggesting its paraphyletic distribution and dispersive genome context result from multiple recombination and gene deletion events.

## Results

### Native Isolation and Purification of Recombinant PS2 S-Layer.

*C. glutamicum* ATCC13032 is widely used in biotechnology and commonly regarded as a reference strain. The strain lacks a 5.97 kb region that contains the *cspB* gene coding for the PS2 S-layer, along with six additional ORFs unrelated to the S-layer biogenesis (32). Analysis of this region revealed the presence of a 7 bp direct repeat that could have led to a recombination event responsible for the loss of these genes compared to S-layer containing strains like ATCC14067. In our work, we used *C. glutamicum* ATCC13032 in which the *cspB* gene (*Cgl2005*) (19) was inserted under its native promoter into the chromosomal *icd* (isocitrate dehydrogenase) locus, hereafter referred to as ATCC13032 *icd::cspB*. This approach allowed us to compare cell phenotype in presence or absence of WT or modified (see below) *cspB* in an otherwise isogenic background. Using SDS-PAGE analysis of surface-extracted proteins, we observed a highly expressed band absent in the wild-type (WT) ATCC13032 strain, which corresponded to the expected size of PS2 (Fig. 1*D*). Negative-stain electron microscopy (ns-EM) inspection of cleared SDS extractions (23) of ATCC13032 *icd::cspB* showed the presence of S-layer-like fragments and sheets (Fig. 1*E*). The power spectrum of the isolated sheets revealed unit cell dimensions of α = β = 171.8 Å and γ = 60°, consistent with previously reported lattice dimensions of PS2 S-layers (20, 33), thus confirming ATCC13032 *icd::cspB* cells expressed and assembled SDS resistant S-layers. An earlier study showed that PS2 devoid of its membrane anchoring C-terminal domain released monomers into the medium and failed to form an organized S-layer, although proteolytic removal of the C-terminal domain from preassembled WT PS2 S-layers did not disrupt the 2D crystal organization (23). To further explore whether the C-terminal domain is necessary for S-layer assembly, we cloned the assembly domain (AD) of PS2 (PS2^AD^; residues 30 to 483) in which we replaced the membrane anchoring domain by a hexahistidine tag and overexpressed in the cytoplasm of *E. coli*. Following cell lysis and centrifugation, we observed a pelleted fraction with a gel-like consistency, composed of PS2 as assessed by anti-His western blotting (Fig. 1*F*). ns-EM confirmed the presence of PS2 S-layer fragments with identical lattice parameters as the ex vivo S-layer (Fig. 1*G*). We next expressed the full-length variant (PS2^FL^) in the *E. coli* cytoplasm (Fig. 1*F*), resulting in an insoluble fraction with PS2 lattice characteristics, but associated with vesicle-like structures (Fig. 1*G*). This suggests that the presence of the MA domain results in a lipid-binding characteristic, consistent with previous observations where the PS2^MA^ domain is responsible for the interactions with the mycomembrane (24).

### Ex Vivo Cryo-EM Structure of the PS2 S-Layer from *C. glutamicum*.

**Fig. 2.**
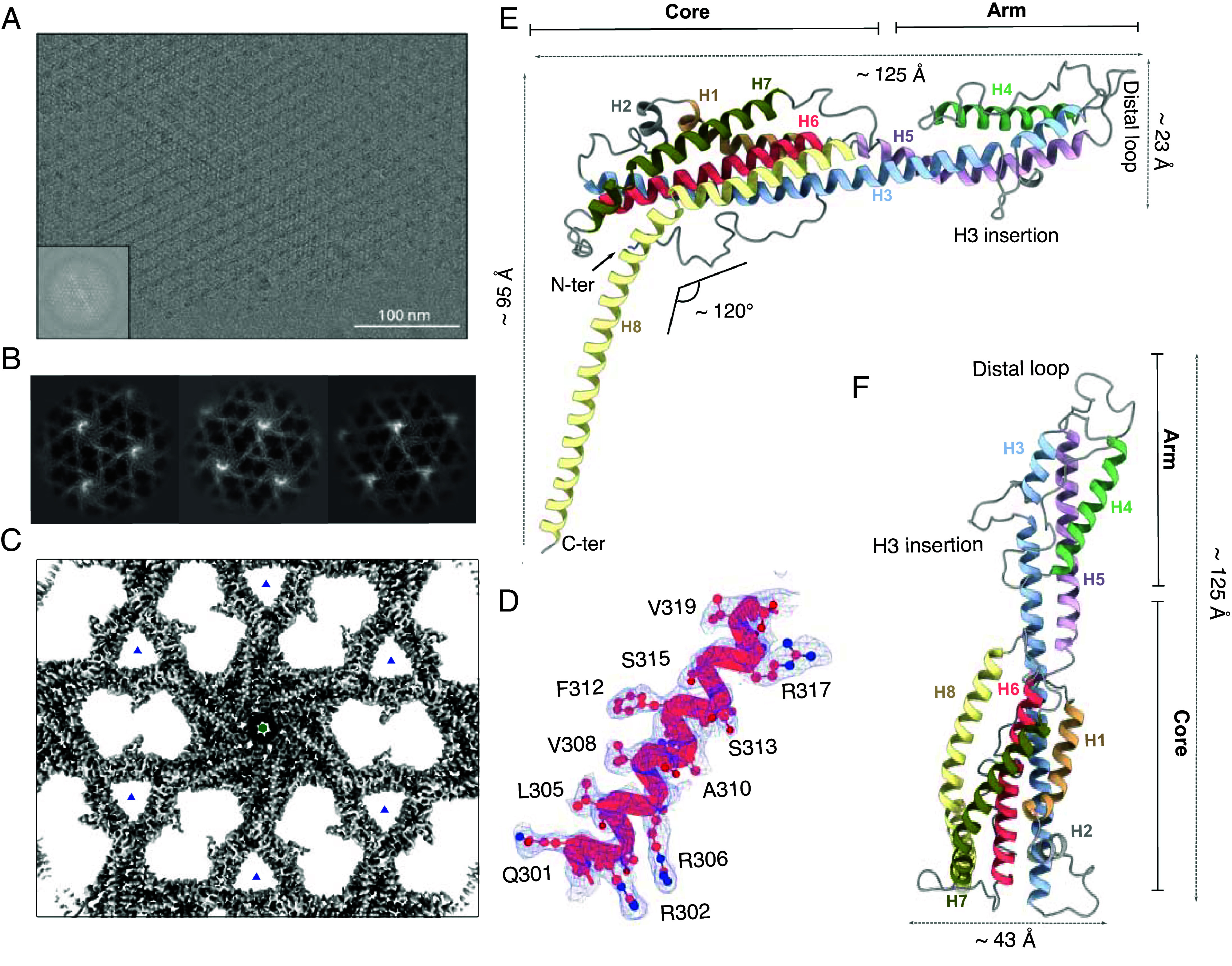
Cryo-EM structure of the ex vivo PS2 S-layer of *C. glutamicum*. (*A*) Raw representative Cryo-EM image of a monolayer PS2 S-layer fragment. (Scale bar, 100 nm.) (*Inset*) Fast Fourier Transform (*B*) Example of 2D class averages of PS2 lattice used for cryo-EM reconstruction.(*C*) Electron density map after EMReady treatment of the PS2 S-layer shown from the *Top* with six-fold symmetry. The average resolution of the map is estimated at 2.51Å. Hexagonal symmetry (green hexagon) and trimeric symmetry (blue triangle) axes are marked. (*D*) Representative density fit of the PS2, showing a fragment of H6. (*E* and *F*) The atomic model of the PS2 monomer, as found in the PS2 lattice, is shown in ribbon representation. PS2 is an all-helix structure, with each helix individually colored. The PS2 monomer can be divided into two regions: the “core” and the “arm.” The core forms part of the hexameric interface, while the arm forms part of the trimeric interface. The distal loop and H3 insertion loop are shown.

**Fig. 3.**
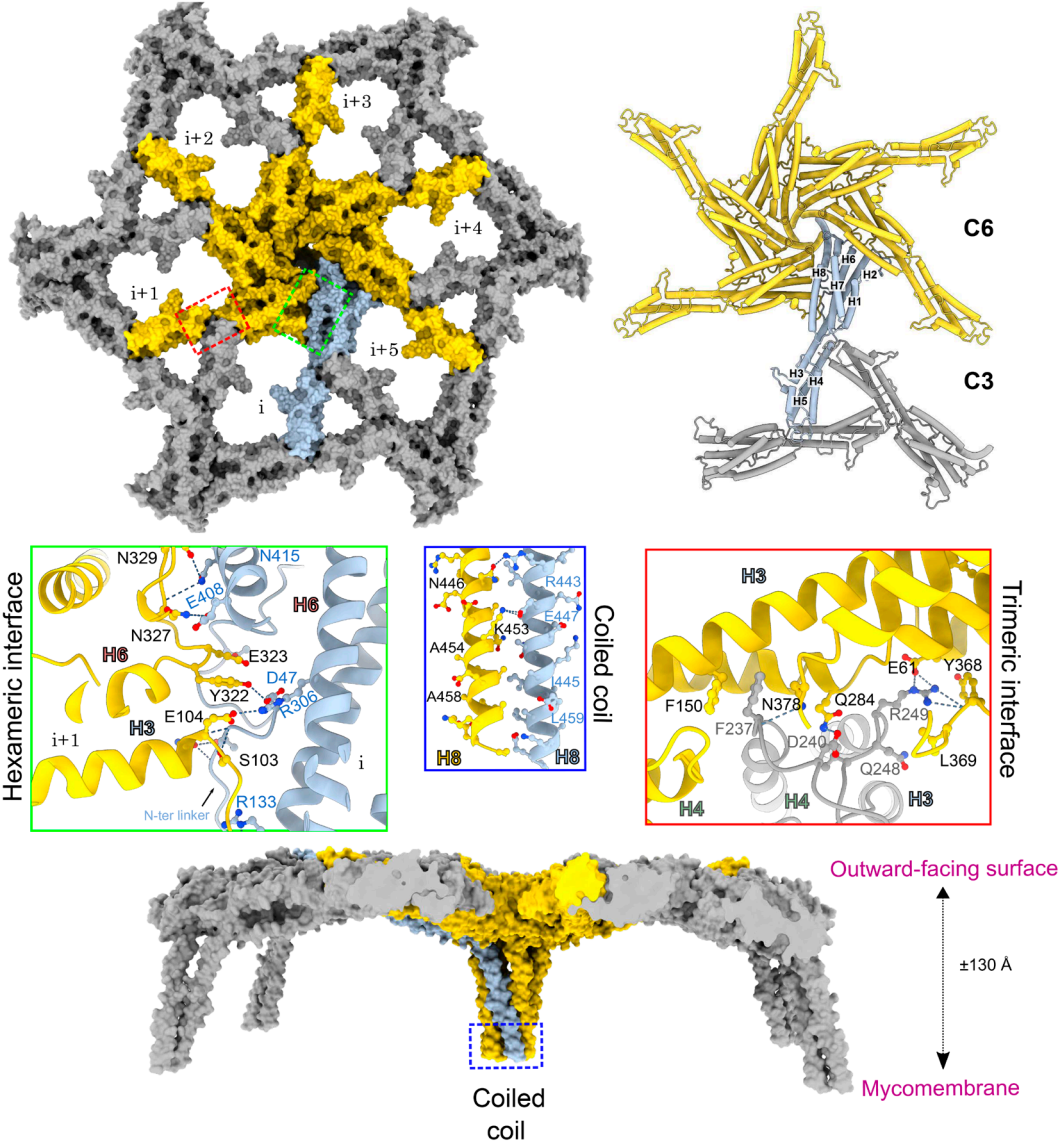
Molecular details of the PS2 lattice assembly. The atomic model of the PS2 S-layer architecture displayed in surface representation in *Top* (*Upper Left*) and side view (*Bottom*), and with protomers in the C6 hexamer and C3 trimer shown in secondary structure representation (*Upper Right*). Protomers in the central hexamer are highlighted in yellow, with one monomer colored in blue, while protomers of adjacent interacting hexamers are shown in gray. The S-layer lattice is stabilized by three distinct protein–protein interfaces: the trimeric (C3) and hexametric (C6) interfaces, boxed in red and green, respectively, and the coiled-coil interface involving H8, boxed in blue. Zoomed-in views display the atomic details of each interacting interface, highlighting hydrogen bonds as dotted blue lines and revealing the specific residues involved in stabilizing each interface.

To obtain high-resolution structural information on the PS2 S-layer, we purified ex vivo S-layer fragments from *C. glutamicum* ATCC13032 *icd::cspB* (*Methods*). Cryo-EM micrographs of the isolated S-layer predominantly displayed top views of single 2D sheets with planar hexagonal symmetry (Fig. 2*A*). Determining S-layer structures by cryo-EM represents a challenge as side views are scarce or sometimes nonexistent, depending on the size of the S-layer fragments. Given the absence of clear side views, we collected tilted images at angles of 15° and 30° allowing for a 3D reconstruction by leveraging the C6 symmetry. The resulting 2D class averages revealed a central hexameric core with six arms, each one extending to two other hexamers, forming a trimeric interface (Fig. 2*B*). Using single-particle cryo-EM workflow with C6 symmetry, the resulting map reported an average resolution ranging from 2.5 Å to 3.8 Å for various orientations. This difference in resolution is attributed to the uneven representation of views, with the lowest resolution orthogonal to the S-layer plane, corresponding to the underrepresented side views (*SI Appendix*, Fig. S1). Despite residual missing wedge artifacts along the Z-axis, the reconstructed map (Fig. 2*C*) allowed unambiguous docking of the AF2 model of PS2 and further manual refinement in real-space to complete the atomic model (Fig. 2*D* and *SI Appendix*, Fig. S2; PDB and EMDB accession codes: 9GK2 and 51414) (34, 35). A single PS2 protomer is composed of eight α-helices adopting an overall banana shape that can be divided into a core and an arm (Fig. 2 *E* and *F*). The core consists of a 28-residue-long N-terminal random coil and six α-helices tightly packed against each other (H1, H2, H3, H6, H7, H8). Helix H8 extends beyond the core and kinks downward with an angle of ~120°, where, together with the other five protomers, they form a hexameric helical funnel. In our structure, we observe clear density until residue A463, leaving the 47 C-terminal residues, which include the predicted membrane anchoring domain, unresolved in the cryo-EM map. AlphaFold predictions for this domain suggest the formation of an alpha helix spanning approximately 3.6 nm (*SI Appendix*, Fig. S2). In *C. glutamicum*, the mycomembrane thickness is estimated to be around 5 nm (36) suggesting that the C-terminal domain would be fully embedded within the membrane. The arm region consists of H3, H4, and H5 helices tightly packed by hydrophobic interactions, and connected by two long linkers: one spanning 27 residues and linking H3 to H4, and another of 16 residues forming a distal loop connecting H4 and H5. Notably, H3 is the only helix that contributes to both the core and the arm regions, featuring an insertion by a 10-residue loop (H3 insertion). The atomic model of the PS2 S-layer shows that the assembly plane is composed of two distinct interfaces (Fig. 3). A C6 hexameric interface where six protomers interact to form the central hexamer, and a C3 trimeric interface formed by the arms of three distinct hexamers in an arm-over-arm arrangement (Fig. 3). This configuration aligns with the previous Saxton and Baumeister classification as an M_6_C_3_ S-layer (20, 37). The side view reveals that from each hexamer, six long α-helices extend downward, forming a coiled-coil bundle with a conical shape (Fig. 3). The PS2 S-layer is stabilized by two extensive protein–protein interfaces that make up the C6 contact of the PS2 core and H8 helix, and C3 contacts of the arms. The intrahexameric interface encompasses a total interaction area of 2,371 Å^2^, involving the C-terminal H8 helix forming a coiled-coil with hydrophobic knobs-in-holes interactions encompassing 631 Å^2^ (Fig. 3); and the N-terminal tip of the core 6-helix bundle (i.e. loops H2-H3 and H6-H7) that docks into a large cleft formed in the side of the core region [i.e. H8 and N-terminal coil; (1,737 Å^2^)] of the neighboring protomer, at a 60° angle (Fig. 3). This interaction predominantly involves hydrophilic contacts, including 26 hydrogen bonds and 6 salt bridges. The interhexameric contacts are driven by the arm regions and form the C3 trimeric interface. This interface involves the distal loop of one protomer that docks into the cleft formed at the limit between the core and arm regions. This contact presents a total surface area of 1,097 Å^2^ involving both hydrophobic and polar interactions. Notably, the distal F237 docks into a hydrophobic pocket interacting with F150 from the other protomer, this type of interaction appears to be conserved across several PS2 variants, as many species contain phenylalanine or tryptophan at this position (*SI Appendix*, Fig. S3). Taken together, the PS2 S-layer is stabilized by a vast network of noncovalent lateral interactions, including hydrophobic contacts, hydrogen bonds, and salt bridges, contributing to its stability.

### PS2 S-Layer Properties and Functional Aspects.

**Fig. 4.**
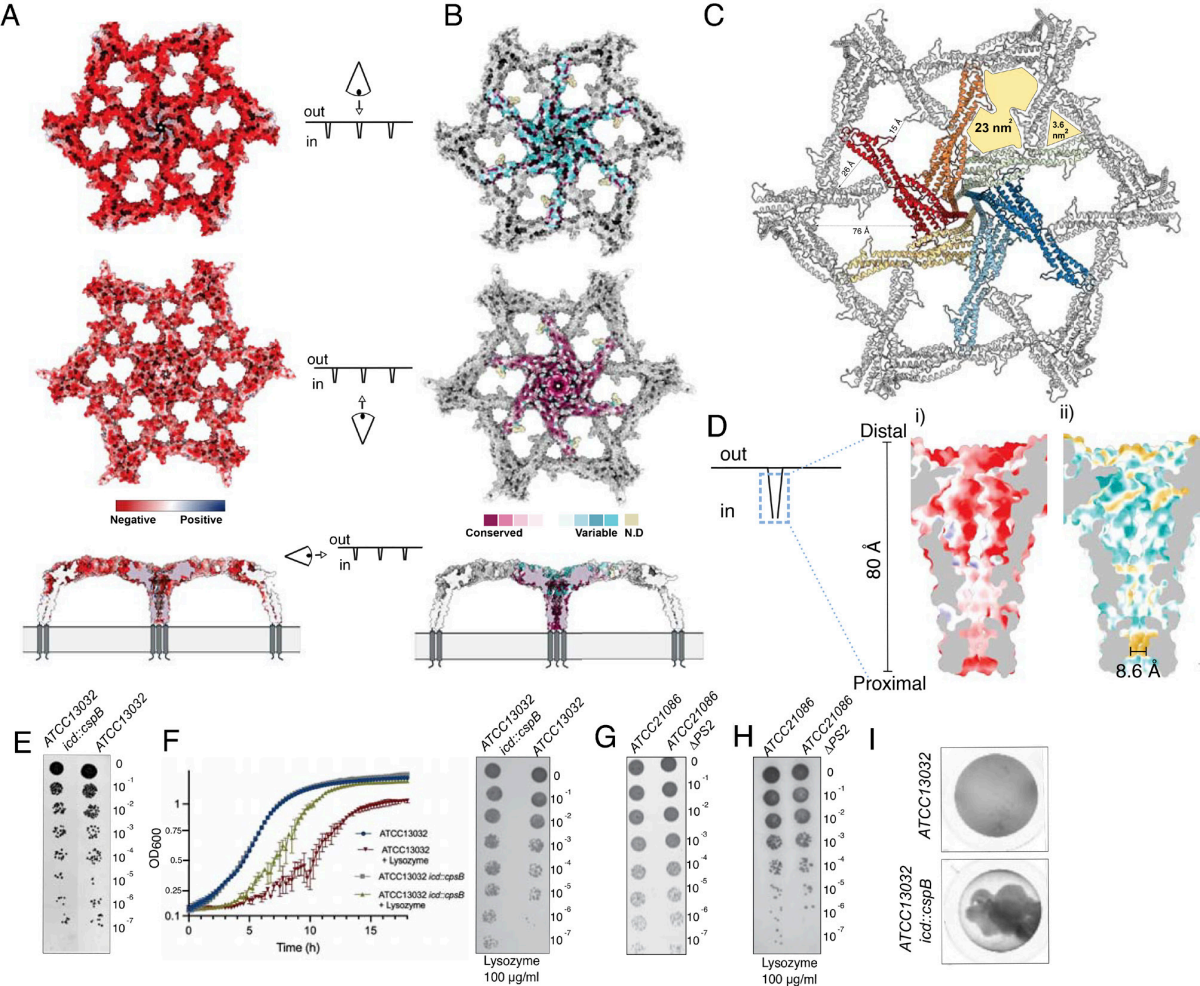
PS2 S-layer is negatively charged and forms large pores. (*A*) PS2 hexamer colored according to its charge distribution (positive and negative charges are colored in blue and red, respectively), shown from extracellular (*Top*), intracellular (*Middle*), and side (*Bottom*) views. (*B*) Conservation of the PS2 S-layer mapped on the hexamer (low conservation in blue to high conservation in purple). The extracellular surface (*Top*) is mainly variable, while the intracellular-facing surface (*Middle*) and the inner channel-like (*Bottom*) show higher levels of conservation. (*C*) Overall arrangement of the pores in the PS2 S-layer seen from the *Top*. Two main pores are highlighted with yellow filling formed upon S-layer assembly as formed by the C3 trimeric interface and C6 hexameric interface. Areas and distances are indicated in Å. (*D*) Cross-section of the channel-like structure formed by the H8 coiled-coil, with the lumen colored according to charge distribution (*i*) (as in panel *A*) and hydrophobicity (*ii*) from hydrophobic regions in yellow to hydrophilic regions in blue. The narrowest constriction of the pore is indicated. (*E*) CFUs count after osmotic downshock comparing the strain with and without S-layer. (*F*) Growth curves (*Left*) comparing ATCC13032 *icd::cpsB* [expressing PS2 (gray and yellow)] with ATCC13032 (red and blue) in the presence (yellow and red) or absence (gray and blue) of 100 μg/mL lysozyme. Growth curve data are sample mean ± s.d., representative of n = 3 biological experiments. Lysozyme was added at the start of the measurements. Lysozyme sensitivity assay (*Right* panel) after incubating ATCC13032 and ATCC13032 *icd::cpsB* in 100 μg/mL of lysozyme overnight. Samples were normalized to an OD_600_ of 0.5, serially diluted 10-fold, and spotted onto an LB agar plate. (*G*) CFUs count after osmotic downshock comparing ATCC 21086 and its *ΔPS2* mutant. (*H*) CFU titers after 8 h of lysozyme treatment (100 μg/mL). (*I*) Different sedimentation properties are observed when ATCC13032 expresses the PS2 compared to its absence. This is manifested from imaging a single well in a 96-well plate after overnight growth, followed by 12 h of incubation at room temperature without shaking.

Analysis of the electrostatic potential reveals that PS2 is a highly negatively charged protein with 18.7% of its residues being Asp and Glu (pI = 4.21) and located at the protein’s surface (Fig. 4*A*). We noticed that the outward-facing surface of the S-layer is more negative than the mycomembrane-facing surface, an observation that seems to be recurrent in bacterial S-layers (38, 39). Many bacterial S-layer proteins require structural metal ions for assembly, usually calcium (11, 38, 40).

Our cryo-EM map does not suggest the presence of glycosylated residues nor the presence of metal ion binding sites in PS2, although the resolution does not allow a fully unambiguous assessment of the latter. To assess whether calcium or other divalent ions are required for PS2 stability, we incubated ex vivo and recombinant PS2^AD^ sheets with 10 mM EDTA. ns-EM analysis showed the presence of S-layers, suggesting that divalent metal ions are not required for PS2 stability in preassembled S-layers (*SI Appendix*, Fig. S4*A*). Additionally, after unfolding recombinant PS2 S-layers with 8 M urea, isolation of monomers, and refolding in the presence of 10 mM EDTA, ns-EM revealed the presence of S-layer fragments (*SI Appendix*, Fig. S4*B*), showing that divalent ions are not essential for either S-layer assembly or stability in vitro.

S-layers often show low levels of sequence conservation even within the same phylogenetic group. We analyzed the sequence conservation of PS2 and mapped it onto the structure. The external and mycomembrane facing surfaces of the S-layer show increased sequence variation or increased conservation, respectively (Fig. 4*B*). The positive selection for variation on the external face aligns with the concept that S-layers might evolve rapidly to adapt to new ecological niches or defend against emerging environmental threats. The most conserved part of the protein maps to the C6 hexameric interface and involves the proximal region of the core (H2-H3 linker) and the docking cleft (*SI Appendix*, Fig. S5). Similarly, residues that contribute to the trimeric interface are conserved suggesting that the overall architecture and assembly mechanism is the same across different PS2 S-layers. The extended arrangement of PS2 results in two major pores in the lattice (Fig. 4*C*). The triangular pore has a height of 26 Å and an area of 3.6 nm^2^. The largest pore is formed by the interaction of two different hexamers with a maximum diameter of 76 Å with a total area of 23 nm^2^. This pore shows a constriction of 14.8 Å due to the presence of the H3 insertion loop (Fig. 4*C*). Finally, a small funnel-like pore is formed by the H8 coiled-coil at the center of the hexamer, possibly extending into a channel through the mycomembrane. This channel-like pore with a height of ~80 Å shows a lumen that is mainly negatively charged but becomes more neutral proximal to the cell, where a belt of leucines (L459) and isoleucines (I455) forms a hydrophobic constriction of 8.6 Å in diameter, the narrowest point in the channel (Fig. 4*D*). As such, the PS2 S-layer can be viewed to represent a continuous semipermeable layer anchored into the mycomembrane on ~8 nm high pedestals, with plausible roles as selectivity and/or barrier and/or support structure (Fig. 4 *A* and *B*). One of the primary functions of the cell envelope is to protect cells from turgor pressure. To test whether the presence of an S-layer increases resistance to osmotic shock, we exposed both strains (with and without S-layer) to an osmotic downshock in water for 45 min and then assessed viability by plating CFUs. The results showed no significant differences between the two strains, indicating that the cell envelope without an S-layer remains fully functional in supporting osmotic pressure (Fig. 4*E*). We then decided to investigate whether the S-layer could provide additional structural support when the peptidoglycan layer is compromised. To test these hypotheses, we performed a lysozyme susceptibility test, a standard method for assessing cell wall integrity. Growth curves of ATCC13032 and ATCC13032 *icd::cspB* showed no difference in the absence of lysozyme, suggesting that S-layer expression does not affect fitness under laboratory conditions. However, upon lysozyme addition, the culture of the strain lacking the S-layer (ATCC13032) showed reduced growth rate (Fig. 4*F*). To assess the generality of this phenotype, we repeated these experiments in a strain that naturally expresses PS2 and compared it to its *ΔPS2* mutant. Specifically, we used the strain *Brevibacterium lactofermentum* ATCC 21086 (later reclassified as *C. glutamicum*) in which PS2 was originally identified (19). SDS-PAGE (*SI Appendix*, Fig. S6*A*) and 2D electron diffraction analysis (*SI Appendix*, Fig. S6*B*) of culture extracts confirmed the presence of PS2 S-layers with isomorphous unit cell parameters in WT ATCC 21086, and the loss of PS2 in the knockout mutant. Both ATCC 21086 and its ΔPS2 mutant exhibited similar growth patterns in rich media (*SI Appendix*, Fig. S6*C*) and comparable resistance to osmotic shock, as assessed by viability measurements using CFU plating (Fig. 4*G*). In presence of lysozyme (100 µg/mL), however, the ΔPS2 mutant showed diminished growth rate compared to WT (*SI Appendix*, Fig. S6*C*), and reduced CFU counts under osmotic shock (Fig. 4*H*). Thus, in both backgrounds, the presence of the PS2 S-layer shows an increased ability to withstand cell envelope destabilizing conditions.

These results align with previous findings on ATCC13869 (41), another strain naturally expressing PS2, and mirrors a similar result observed in *C. difficile* (39, 42). Given that lysozyme is a small protein with a hydrodynamic radius of 1.9 nm and therefore expected to be capable of passing through the PS2 pores, we hypothesize that the partial protection from the S-layer may result from its adsorbing lysozyme molecules due to the positively charged surface of lysozyme (pI=9.3) and/or acting as a mechanical support that helps the cell envelope maintain turgor pressure, as seen for *B. anthracis* S-layers (8, 10, 11). Finally, another phenotypic difference observed while growing ATCC13032 with and without PS2 expression was the variation in coagulation and sedimentation properties. Unlike its WT parent, the ATCC13032 *icd::cspB* strain expressing PS2 exhibited strong flocculation under static growth conditions (Fig. 4*G*). Thus, the presence of the PS2 S-layer appears to alter the surface properties of *C. glutamicum*, resulting in an increased coagulation of cells, a property that may also impact biofilm formation and adhesion to surfaces (43).

### Engineering and Biogenesis of the S-Layer in *C. glutamicum*.

**Fig. 5.**
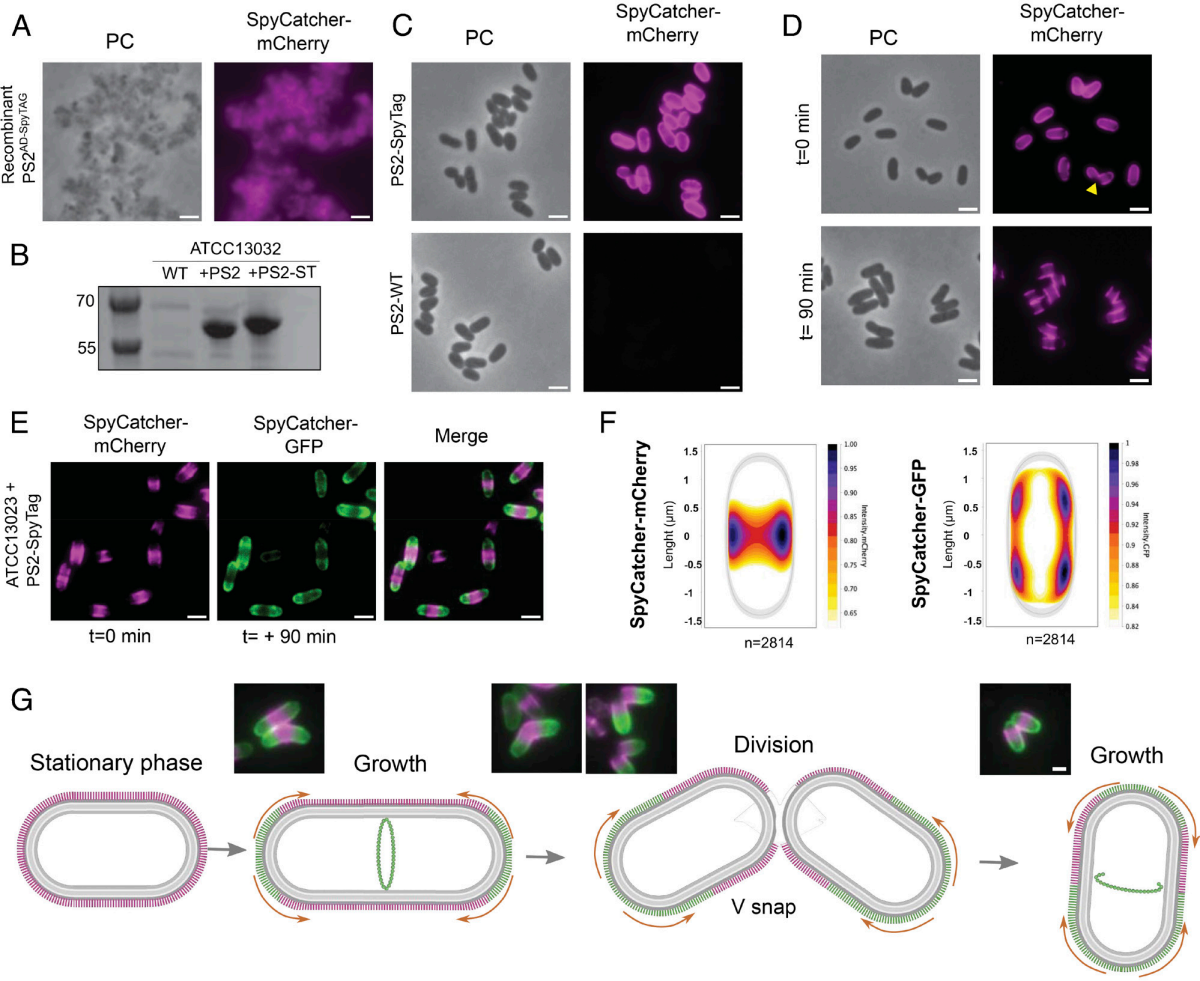
Engineering of the PS2 S-layer and polar assembly. (*A*) Micrograph of recombinant (*E. coli* expressed) PS2^AD-SpyTAG^ incubated with Spycatcher-mCherry. (*B*) Coomassie stained SDS-PAGE of the extracted cell surface protein comparing ATCC13032 and transformed strain expressing PS2 and PS2^SpyTAG^. The strain expressing the SpyTag shows an increased molecular weight. (*C*) Micrograph of *C. glutamicum* expressing PS2^SpyTAG^ (*Top*) or WT PS2 (*Bottom*) and incubated with SpyCatcher-mCherry. (*D*) Micrograph of Spycatcher-mCherry stained PS2^SpyTAG^ at t = 0 min and t = +90 min. At a later point, the old (stained) S-layer is restricted to the middle of the cell with no apparent diffusion. After cell division (V-snap), two new poles are formed deprived of S-layer (yellow arrowhead). (*E*) Representative micrograph of PS2^SpyTAG^ expressing strain first labeled with Spycatcher-mCherry and pulse chased with a second stain using Spycatcher-GFP after 90 min. The new S-layer (green) is inserted at the poles. In all of the above panels, (Scale bar, 2 µm.) (*F*) Normalized heat map representing the localization pattern of SpyCatcher-mCherry (old S-layer) and SpyCatcher-GFP (new S-layer). A total of 2,814 cells were analyzed. The images are representative of experiments made independently in triplicate. (*G*) Model of the S-layer dynamics during growth and division. During the stationary phase, the cell is fully covered by a continuous S-layer (shown in pink). Upon entering the growth phase, the elongasome assembles at the poles to drive polar peptidoglycan (PG) synthesis and cell envelope assembly, while the divisome assembles at midcell, guided by the FtsZ ring. New S-layer material (green) is synthesized at the poles, coinciding with PG synthesis. Orange arrows indicate the direction of S-layer expansion as new material is added. During daughter cell separation (V-snapping), the newly exposed pole is initially devoid of the S-layer (i.e. panel *D*, yellow arrowhead). Representative cells of each stage are shown above. (Scale bar, 1 µm.)

S-layers are attractive biomaterials with interesting properties such as regular autoassembly. We have engineered the PS2 S-layer of *C. glutamicum* to display the SpyTag, which forms a covalent bond with SpyCatcher-tagged proteins (44). Structural analysis revealed that both termini of PS2 are not surface exposed suggesting that the SpyTag should be added internally. Guided by the structure of the S-layer lattice, we introduced the 18 residues that form the SpyTag in the H3 insertion loop. This loop is absent in most of the PS2 sequences except for *C. glutamicum* strains ATCC13870 and ATCC14068. In addition, sequence analysis showed that the H3 insertion loop is composed of a sequence repeat at the DNA level resulting in the amino acid sequence: SINPDGSINPD, suggesting that it arose from a small duplication event. Therefore, we inserted the SpyTag in position 168 (PS2^SpyTag^) (*SI Appendix*, Fig. S7). To test that the insertion is functional and does not impact PS2 self-assembly, we purified from *E. coli* the recombinant PS2^AD-SpyTag^ and mixed it with 1.5 molar excess of SpyCatcher-mCherry. Fluorescence light microscopy showed the presence of fluorescent recombinant S-layers (Fig. 5*A*). As a control, we used the WT version (PS2^AD^) which did not exhibit any fluorescent signal (*SI Appendix*, Fig. S8*A*). ns-TEM confirmed that the addition of SpyTag in complex with SpyCatcher-mCherry does not alter the formation of ordered 2D sheets (*SI Appendix*, Fig. S8*B*). These observations indicate that engineered PS2^SpyTag^ is functional and allows for specific binding of a SpyCatcher fusion protein. Next, to engineer PS2 full-length in vivo, we cloned the PS2^SpyTag^ under its native promoter into the pTGR5 shuttle plasmid (45) (Addgene ID 237443) (46). We expressed this construct into the S-layer lacking strain (ATCC13032) and validated its expression (Fig. 5*B*). To explore in vivo labeling of the PS2 S-layer, we incubated *C. glutamicum* ATCC13032 expressing PS2^WT^ or PS2^SpyTag^ with SpyCatcher-mCherry and visualized the cells using fluorescence microscopy. While no significant mCherry signal was observed in the strain expressing the PS2^WT^, the PS2^SpyTag^ strain presented a bright and uniform fluorescence signal surrounding the cell surface (Fig. 5*C*). cryo-EM imaging of these cells shows PS2^SpyTag^ assembles into a crystalline lattice on the cell surface (*SI Appendix*, Fig. S9). These results demonstrate the potential for engineering PS2 to selectively attach specific proteins to the extracellular surface of *C. glutamicum*.

Next, we used the ability to covalently label in vivo PS2 S-layers to investigate the molecular mechanisms of S-layer biogenesis by means of a two-color pulse-chase experiment with two distinct fluorescent SpyCatcher fusions. Prior studies in gram-positive (30), gram-negative bacteria (31), and Archaea (47) showed that de novo S-layer assembly is localized predominantly at mid-plane, suggesting a colocalization with sites of cell elongation and cell wall synthesis. Interestingly, *Corynebacteriales* grow from their poles, where the cytoskeletal protein DivIVA (also known as Wag31) guides the elongasome and peptidoglycan insertion. The second site of cell wall biosynthesis is the septum, where the FtsZ-guided divisome incorporates the new cell wall (48, 49) (*SI Appendix*, Fig. S10). If S-layer assembly colocalizes with peptidoglycan synthesis, we hypothesized that in *C. glutamicum*, S-layer biogenesis would occur primarily at the poles and/or at mid-cell. To test that hypothesis, we first saturated the surface of *C. glutamicum* ATCC13032 PS2^SpyTag^ with a pulse labeling of SpyCatcher-mCherry and observed that after 90 min of continued growth, the poles were devoid of fluorescence signal (Fig. 5*D*). This result shows that the PS2 S-layer exhibits nondiffusive behavior, as the old, labeled PS2 is not redistributed across the cell surface. Second, these data suggested that new S-layer is incorporated at the poles. To further support this idea, we performed chase labeling of the new, unstained S-layer with SpyCatcher-sfGFP. The images showed that the old S-layer (labeled with SpyCatcher-mCherry) localized over the lateral body, away from the poles, whereas the newly synthesized S-layer (labeled with SpyCatcher-GFP) primarily displayed a polar signal (Fig. 5*E*). As expected from the visual inspection of the data, fluorescence signal quantification of >2,000 dually labeled cells confirmed these patterns (Fig. 5*F*). Thus, our results support the model that new S-layer assembly colocalizes with the zones of polar peptidoglycan synthesis and cell elongation (Fig. 5*G*). Notably, dividing cells show a lack of labeling in the division plane, as well as on the new cell poles during the V-snap and shortly after completion of cell separation (Fig. 5*G*). This suggests the septum and the new poles are temporarily devoid of S-layer, a notion that is reinforced by the observation of cells with a unipolar lack of S-layer during cryoEM imaging (*SI Appendix*, Fig. S9*C*). A lack of S-layer in the division plane was also observed during cryotomographic imaging of *C. glutamicum* thin sections by Isbilir et al. (36).

### Phylogenetic Analysis of PS2.

The available structures of bacterial S-layers show a lack of structural homology across genera (8, 38, 39, 50, 51) indicating that S-layers have arisen independently multiple times throughout evolution (33). Here, we set out to study the phylogeny and distribution of PS2 in the order *Corynebacteriales* (*SI Appendix*, *Methods*). Our analysis revealed that PS2 homologues are exclusively found in *Corynebacterium*, suggesting genus specificity. However, its presence is sporadic, with only 102 hits (i.e. cutoff of e-value 1e-05 to ATCC13032 PS2 –GenBank sequence AAX43986.1) out of the 2,325 genomes analyzed (4.25%) (*SI Appendix*, *Methods* and Fig. S11*A*). PS2 genes show a scattered, paraphyletic distribution across *Corynebacterium* species, sometimes specific to just some strains within the same species. This is the case for *C. glutamicum*, where the reference strains ATCC4067 and ATCC13032 respectively hold or lack the *cspB* gene. The presence of a 7 bp sequence repeat, an integrase and an IS element in the genomic region encoding *cspB,* suggests that a recombination event may have resulted in the acquisition or deletion of *cspB* in these strains (32). Moreover, the paraphyletic distribution of *cspB* could be the result of repetitive losses of *cspB* or acquisitions through horizontal gene transfer. To discriminate between these two scenarios, we studied the genomic context of the *cspB* gene. The result of this analysis showed that *cspB* presents varying genomic contexts across different species, whereas it seems to be conserved within related species (*SI Appendix*, Fig. S11*B*). Next, we compared the genomic loci where *cspB* is present with those of closely related species lacking PS2 (*SI Appendix*, Fig. S12). Our analysis revealed many different scenarios including a substantial number of deletions, insertions, and even inversions surrounding the *cspB* gene. These genomic alterations sometimes affected only the *cspB* gene itself or involved neighboring genes, suggesting that *cspB* gene resides within or is linked to mobile genetic regions. Nevertheless, the phylogenetic tree of the *cspB* gene appears to largely follow the full genome phylogeny of the *cspB* positive strains (*SI Appendix*, Fig. S13), suggesting that the paraphyletic distribution and dispersed genomic context of PS2 are likely a result of multiple recombination and gene deletion events.

## Discussion

Actinobacteria are gram-positive bacteria characterized by a complex, diderm cell envelope, composed of the cytoplasmic membrane (IM), peptidoglycan layer (PG) with attached arabinogalactans (AG), and a specialized outer membrane (OM) composed of mycolic acids (Fig. 1*A*). The common industrial production species *C. glutamicum* adds an additional layer of complexity by anchoring a continuous protein surface layer (S-layer) atop the OM. Here, the 3D cryo-EM structure of extracted PS2 S-layer fragments from *C. glutamicum* reveals this S-layer as a continuous, semiporous monolayer of C6:C3 symmetry and 25 Å thickness, with a regular network of gaps of ~26 and ~76 Å maximum diameter (~36 and ~230 Å^2^ surface area, resp.; Figs. 2*B* and 4*C*). This monolayer is formed by PS2 hexamers with protruding arms that maintain C3 contacts with neighboring hexamers (Fig. 3). The PS2 hexamers are anchored in the mycomembrane by the C-terminal ~27 residues of the protein (absent in the reported structure), which are found at the end of a funnel-like coiled-coil of ~80 Å height and formed by the elongated H8 helix. As such, PS2 hexamers attain a parasol-like structure (Fig. 4*A*), resulting in the formation of a 7 nm pseudoperiplasmic space atop the mycomembrane. The functional significance of this pseudoperiplasmic space, or indeed the PS2 S-layer, remains largely unknown.

In the presence of the S-layer, we find *C. glutamicum* became less sensitive to extracellular lysozyme. We hypothesize this protective activity of the PS2 S-layer may be the result of two main possible factors: 1) the semiporous S-layer may have a barrier activity that influences the effective dose that reaches the cell wall, or 2) the cell envelope destabilizing activity of the lysozyme exposure may be mitigated by the S-layer. When considering PS2 as a diffusion barrier, we note that most lytic enzymes, like lysozyme, would need to pass at least the mycomembrane to reach their targets. Even so, high concentrations of lysozyme (50 to 500 µg/mL) have been found to reach and degrade the peptidoglycan layer by an unknown mechanism (52). The porous structure of the PS2 lattice is compatible with the passage of proteins of up to 50 to 100 kDa [i.e. ~50 to 60 Å diameter when considered spherical and average density of 1.35 g/cm^3^; (53), making it unlikely that PS2 would act as a physical barrier to most lytic enzymes including lysozyme (14.3 kDa). However, in addition to size-based limitations, the highly negative charge of the S-layer could also electrostatically exclude or sequester charged solutes. In the case of lysozyme, its net positive charge (pI=9.3) could result in it getting adsorbed at the S-layer by means of electrostatic interactions, thereby reducing the effective concentration of lysozyme in the medium. Whether such steric or electrostatic occlusions are at play in the reduced lysozyme susceptibility of PS2 expressing cells is currently unclear. Furthermore, although we observe high S-layer coverage in this study, it should be noted that depending on strain, growth phase, and growth medium, *C. glutamicum* can show variable coverage in S-layer, resulting in a patchy appearance on the cell surface (36, 54). It is unclear how a more patchy S-layer distribution will influence its (partial) protective activity. As an alternative or synergistic mechanism, the S-layer may act as a mechanical support structure to the cell envelope, thereby mitigating envelope destabilizing conditions such as cell-wall lytic and/or osmotic challenges. Such mechanical support activity is considered a primary function of archaeal S-layers (53), and also in *Bacillus anthracis* recent observations are in agreement with the Sap and EA1 S-layers adopting a cell envelope supportive role (10, 11). Genetic loss of these S-layer proteins, or an induced loss of the lattice structure resulted in compromised tensile properties of the bacterial cell envelopes, and in an increased susceptibility to hypo-osmotic conditions.

Whether or not adopting a barrier or mechanical function, it can reasonably be expected that the secretion and assembly of cell envelope components, including S-layers, need to be coordinated with cell growth and division. These cellular processes are orchestrated by cytoskeletal proteins that act as recruitment signals and provide the dynamics of cell cycle progression. The mechanism by which the S-layer assembly expands in coordination with the entire cell envelope remains unclear. This is particularly puzzling given that S-layers form regular lattices, which typically grow by the addition of subunits at their edges. Therefore, unless the S-layer is composed of a mosaic of crystalline microdomains or the cells are capable of dynamically assembling and disassembling the lattice, the S-layer would be expected to associate with the cell envelope as a continuous unit, with lattice edges exposed only in regions of cell expansion. Only recently, a handful of studies have shed light on the dynamics of the S-layer during cell growth. In bacteria, this process has been studied in *C. crescentus* (31) and *Clostridioides difficile* (30), revealing that S-layer growth occurs mainly at mid-cell, indeed coinciding with the regions where new peptidoglycan is inserted by the divisome machinery. In *C. crescentus*, inhibition of MreB (a major cytoskeletal component that drives the elongasome) resulted in delocalized S-layer insertion (29). Corynebacteriales lack MreB and instead contain the coiled-coil elongasome scaffold DivIVA, which is responsible for their rod shape and involved in localizing the peptidoglycan synthesis machinery to the poles (17). Other components of the Corynebacteriales envelope, such as the arabinogalactan and mycolic acids, also show polar assembly dynamics (55, 56).

Our study examines S-layer biogenesis in polar-growing bacteria, revealing that this process occurs exclusively at the poles. We did not observe new S-layer being added at mid-cell where divisome-driven peptidoglycan synthesis also takes place, indicating that S-layer assembly is only associated with the elongasome (Fig. 5*G*). Previous models for S-layer assembly suggest that cell wall expansion is a driving force in cell envelope growth and a predictor of local S-layer biogenesis, where a pool of free S-layer proteins (SLPs) exists in the cell wall to plug S-layer-free regions (29–31). If this was true for *Corynebacterium*, we would also observe labeling of the new S-layer at mid-cell, at least in cells initiating division. During advanced cell division, *C. glutamicum* daughter cells exhibit a V shape, which is believed to result from the mechanical fracture of the rigid envelope layers. V-snapping occurs rapidly (within 10 mins) (57) and leads to the exposure of a newly formed cell pole, that subsequently becomes a site for cell wall elongation. In our images, we captured V-snapping events where the newly exposed poles lack an S-layer, and only become labeled upon full separation of the daughter cells (e.g., Fig. 5 *D* and *G*). Little is known on how the elongasome works in *Corynebacteriales* or how and when it is assembled at the newly formed poles. Our observations find no evidence for new S-layer assembly at the division plane, and are more compatible with a model where S-layer growth occurs only after division is completed and the cells resume elongation. This would suggest that S-layer secretion and/or biogenesis are directly or indirectly coordinated by elongasome components. Different to most bacterial S-layers, however, the *Corynebacteriales* S-layers are membrane anchored (i.e. the mycomembrane) rather than attached to the cell wall or a secondary cell wall polymer. The PS2 S-layer is exported by means of an N-terminal leader sequence and the SEC translocon. How and where it traverses the mycomembrane to reach the cell surface is unknown (58). Our demonstration that the recombinant introduction of merely the *cspB gene* into ATCC13032 is enough to export and assemble the PS2 S-layer could suggest that PS2 secretion does not require dedicated machinery, but occurs through a preexisting pathway. However, this would need to be tested in other *Corynebacterium* species, as the presence of an *slp* gene does not always correlate with the presence of an S-layer (59). Future studies will be required to identify the mode of secretion, and to evaluate whether and how secretion of PS2 is coordinated by the elongasome. Such studies could focus on i) the localization of the SEC machinery used by PS2 for translocation across the inner membrane (58) ii) tracking new S-layer formation under DivIVA depletion conditions, iii) evaluating S-layer assembly in null mutants of known mycomembrane insertion of translocation pathways and (iv) localizing a monomeric PS2 (assembly-incompetent mutant) to determine whether it diffuses in the cell envelope or exhibits polar localization.

Finally, S-layers have attracted significant interest in bioengineering materials and synthetic biology as a display platform due to their crystalline self-assembly behavior which facilitates precise spatial positioning and high-density material display. Our work demonstrates that recombinant introduction of *cspB* into *C. glutamicum*, and likely other species, readily results in the secretion and assembly of PS2 S-layers. Moreover, we find that the PS2 S-layer alters cell coagulation and flocculation, properties important for fermentation and downstream processing behavior. Furthermore, we show that the engineering of PS2 by insertion of the SpyTag, results in an easy platform for covalent surface display both in vitro and in vivo by means of SpyCatcher-SpyTag and related protein conjugation technologies. Our structural analysis predicts that likely, these same sites (i.e. the H3 insertion loop, residues 162-171) are amenable to the insertion and abundant, regular surface display of larger fusion peptides or whole proteins. As such, we anticipate that this structural work and PS2 engineering technology provide an interesting expansion to the broad use of *C. glutamicum* as an industrial workhorse for the production of (poly)peptides, amino acids, and other fermented materials.

## Methods

### Bacterial Strain and Growth Conditions.

All bacterial strains used in this study are listed in *SI Appendix*, Table S1. *E. coli* DH5α was used for cloning purposes and was grown in LB media or agar plates at 37 °C supplemented with 50 µg/mL kanamycin or 50 µg/mL Ampicillin when required. For protein production, *E. coli* BL21 (DE3) was grown in TB media supplemented with 50 µg/mL Ampicillin at the appropriate temperature for protein expression. *C. glutamicum* ATCC13032 was used as a WT strain and *C. glutamicum* ATCC13032 *icd::cpsB* expressing the *cspB* gene (Cgl2005) (19). *C. glutamicum* strains were grown in LB or BHI media at 30 °C and 120 rpm and were supplemented with 25 µg/mL kanamycin and/or 20 mL/L of sodium lactate when required to induce higher levels of PS2 expression (54).

### Cloning for Recombinant Production in *E. coli*.

The *cspB* gene (Uniprot ID: Q04985) coding from residues 30 to 510 was amplified by PCR using oligos p849 and p868 whereas the assembly domain construct (PS2^AD^) (residues 30 to 483) was amplified with oligos p849 and p850 using as a template the gDNA of the *C. glutamicum* ATCC13032 *icd::cpsB*. The PCR fragments were cloned into a linearized pASK-IBA3plus vector (using primers p321 and p322) by Gibson assembly leading to plasmids A232 and A231 and transformed into chemically competent DH5α *E. coli* (New England BioLabs). PS2-spyTAG versions were produced by site-directed mutagenesis using oligos p873 and p874 leading to plasmid A235. SpyCatcher-GFP and SpyCatcher-mCherry were synthetically ordered (Integrated DNA technologies - IDT) and cloned into the linearized pASK-IBA3plus vector leading to plasmids A233 and A234 respectively. Plasmids were sequence verified (Eurofins). All plasmids and primers are listed in *SI Appendix*, Table S1.

### Cloning for Recombinant Protein Expression in *C. glutamicum*.

For ectopic expression of PS2 variants in *C. glutamicum* ATCC13032, we amplified the *cspB* gene with its native promoter with oligos p864+p865 using as a template the ATCC13032 *icd::cpsB* (which contains the *cspB* gene with its native promoter). PCR fragment was cloned by Gibson assembly into a linearized pTGR5 (using oligos p862+p863) leading to the formation of the A236 plasmid (Addgene ID 237442) (60). Insertion of the SpyTAG was done using oligos p873+p874 and A236 as a template, leading to the A242 plasmid (Addgene ID 237443) (46). Plasmids were sequence verified (Eurofins) and transformed into electrocompetent *C. glutamicum* cells as described in (49). Sequences of interest are found in *SI Appendix*, Table S2.

### Ex Vivo PS2 Purification.

To purify ex vivo PS2 S-layer fragments from *C. glutamicum* ATCC13032 *icd::cpsB*, we grew 500 mL of culture in LB medium with 2% sodium lactate overnight at 30 °C. The culture was harvested by centrifugation (10 min at 5,000×*g*), and the pellet was resuspended in PBS + 1% SDS, followed by a 2-h incubation with shaking. The mixture was then homogenized using a blender and loaded onto a 20% sucrose cushion. After centrifugation (30 min at 4,800×*g*), the layer above the cushion, enriched with S-layer fragments, was recovered while cells were found in the pellet. The S-layer fragments were centrifuged again (30 min at 20,000×*g*) and washed with 100 mM NaCl and 20 mM HEPES pH 7.

### Growth Curves, Lysozyme Resistance, and Osmotic Shock.

All strains were initially plated on LB agar for 2 d at 30 °C. A single colony from each plate was then inoculated into LB media supplemented with 2% sodium lactate and incubated overnight at 30 °C with 120 rpm shaking. The following day, 2 ml of LB media supplemented with 2% sodium lactate were inoculated with the overnight cultures to achieve a starting OD_600_ of 0.05, and 200 μL of each culture was dispensed into individual wells of a 96-well plate. For experiments involving lysozyme, a final concentration of 100 μg/mL was used. The 96-well plates were then loaded into the Cytation One system (BioTek) and incubated at 30 °C with double orbital shaking. OD_600_ measurements were recorded every 15 min. Data analysis and plotting were performed using Prism8 software. To assess resistance to osmotic shock, a single colony was inoculated into LB media supplemented with 2% sodium lactate and incubated at 30 °C with shaking at 120 rpm. When the culture reached the exponential phase (OD_600_ = 0.6), the medium was removed by centrifugation, and the cell pellet was resuspended in an equal volume of deionized water. The cells were then incubated for 45 min at 30 °C with shaking at 120 rpm. Following this, a 1/10 serial dilution was prepared in water, and 2 µL of the diluted suspension was plated for CFU counting. All experiments were conducted in triplicate, and the results are presented as the mean ± SD.

### Protein Expression and Purification.

PS2 and SpyCatcher derivatives were expressed in *E. coli* BL21 (DE3) grown in Terrific Broth (TB) supplemented with 100 µg/mL of Ampicillin at 37 °C and induced with 200 µg/L anhydrotetracycline when OD_600_ reached 0.6. Following induction, the temperature was dropped to 23 °C for overnight expression. Next day, cells were harvested by centrifugation (20 min at 5,000×*g*) and pellets were kept at −20 °C. Cell pellet was resuspended in 100 mL of lysis buffer [50 mM HEPES pH8, 300 mM NaCl, 1 mM MgCl2, DNase, lysozyme, and EDTA-free protease inhibitor cocktails (ROCHE)] at 4 °C and lysed by sonication. The lysate was centrifuged for 60 min at 30,000×*g* at 4 °C. For SpyCatcher derivatives, the cleared lysate was loaded onto a Ni-NTA affinity chromatography column (HisTrap FF crude, GE Healthcare) and washed extensively with buffer A (50 mM HEPES pH8, 300 mM NaCl, 10 mM imidazole). His-tagged proteins were eluted with a linear gradient of buffer B (50 mM HEPES pH8, 300 mM NaCl, 5% glycerol, 1 M imidazole). The eluted fractions containing the protein of interest were pooled, concentrated, and loaded onto a Superdex 75 16/60 size exclusion (SEC) column (GE Healthcare) pre-equilibrated at 4 °C in SEC Buffer (50 mM HEPES pH8, 150 mM NaCl). The peak corresponding to the protein was concentrated, flash-frozen in small aliquots in liquid nitrogen and stored at −80 °C. For PS2^AD^, following centrifugation of the lysate, an additional layer with a gel-like consistency was observed between the supernatant and the pellet, primarily containing PS2. This PS2-containing pellet was carefully collected and subsequently resuspended in SEC buffer supplemented with 1% DDM (n-dodecyl-β-D-maltoside), followed by overnight incubation. The next day, PS2 S-layer mixture was centrifugated at 23,000×*g* for 40 min. The supernatant was discarded and the pellet containing S-layers was resuspended in fresh SEC buffer. This washing step was repeated five more times to ensure the removal of the detergent and contaminants. The purity of the sample was assessed using SDS-PAGE and ns-EM.

### Cryo-EM Sample Preparation and Data Collection.

High-resolution cryo-EM datasets were collected using Quantifoil™ R2/1 300 copper mesh holey carbon grids. Grids were glow-discharged at 5 mA plasma current for 1 min in an ELMO (Agar Scientific) glow-discharger. A Gatan CP3 cryoplunger set at −176 °C and relative humidity of 90% was used to prepare the cryosamples. Just before plunging, DDM to a final concentration of 0.02 % was added to the ex vivo purified PS2 S-layer solution and 3 μL was applied on the holey grid and incubated for 60 s. The sample was backblotted using Whatman type 2 paper for 3 s and plunge-frozen into precooled liquid ethane at −176 °C. High-resolution movies were recorded at 300 kV on a JEOL Cryoarm300 microscope equipped with an in-column Ω energy filter (operated at slit width of 20 eV) automated with SerialEM 3.0.850. The movies were captured with a K3 direct electron detector run in counting mode at a magnification of 60 K with a calibrated pixel size of 0.71 Å/pix, and exposure of 60e/Å2 taken over 60 frames. A total of 15,208 movies were taken, of which 11,988 were measured by tilting the stage at 30° and 3,220 at 15° with a defocus range of −1.1 to −1.6 micrometers. For whole-cell imaging, 3 µL of *C. glutamicum* ATCC13032 expressing PS2-SpyTag at an OD_600_ of 3.0 were applied to freshly glow-discharged Lacey carbon grids (300 mesh Cu, Agar Scientific). Grids were double-side blotted for 3.2 s and plunge-frozen in liquid ethane as described above. Cryo-EM data were acquired as 2 × 2 montages using image shift at a 5 k magnification (10.2 Å per pixel) at a dose of 22.7 e^−^ per pixel per second in counting mode, with a total exposure of 4.99 s. Images were collected with a defocus of −5 to −10 µm.

### Cryo-EM Single Particle Analysis and Structure Determination.

Movies were imported to CryoSPARC (61) where they were motion-corrected using Patch Motion Correction and defocus values were determined using Patch CTF. Exposures were curated and particles were picked using blob picker and extracted with a box size of 600 × 600 pixels. Several rounds of 2D classification were needed in order to clean selected particles providing a set of 1.268.481 high-quality particles and further centered at a hexameric axis. We performed nonuniform refinement using C6 symmetry. Next, we used two rounds of 3D classification in CryoSPARC and selected a single class showing higher-resolution information containing 521.917 particles. Finally, we used reference-based motion correction on those particles and the nonuniform refinement job to generate the final map with an average resolution of 2.44 Å according to the FSC curves (*SI Appendix*, Fig. S1). Finally, we used EMready (62) to improve the interpretability of the map in those regions where resolution was lower and finally, we built the atomic model using a combination of ModelAngelo (63), AlphaFold2 (64) followed by manually rebuilding in Coot (65). A final round of refinement was performed using Phenix (66), and figures were generated using ChimeraX (67). Map and model statistics are found in *SI Appendix*, Table S3. Analysis of the evolutionary conservation of amino acids was performed using ConSurf webserver (https://consurf.tau.ac.il/), using default parameters that included 150 sequences with an ID ranging from 95 to 35% from the UNIREF-90 database.

### Phase Contrast and Fluorescence Microscopy and Image Analysis.

For imaging, a single colony of *C. glutamicum* ATCC13032 or strains expressing different variants of the PS2 S-layer were inoculated in 10 mLof LB media supplemented with 20 mL/L of sodium lactate and with 25 µg/mL kanamycin when required and grown for 5 h at 30 °C. At this point, we added SpyCatcher-mCherry to a final concentration of 50 µM and the culture was grown overnight at 30 °C with 120 rpm shaking. Next day, 10 mL of the overnight culture was harvested by centrifugation at 5,000×*g* for 5 min and washed three times with LB media to finally resuspend the pellet in 3 mL of fresh LB media. In a new culture tube, 2 mL of LB supplemented 20 mL/L of sodium lactate and with 25 µg/mL kanamycin (when required) were inoculated with 500 µL of the above washed overnight culture. The culture was placed at 30 °C for 1 h after which SpyCatcher-GFP was added to a final concentration of 50 µM and incubated for 1 h. For HADA labeling, cultures were incubated with 0.5 mM HADA for 20 min at 30 °C in the dark. Finally, the culture was harvested by centrifugation at 5,000×*g* for 5 min washed three times with 0.9% NaCl and diluted to OD_600_= 0.05 and 3 µL was transferred to the LB-agar strip. Images were collected in phase contrast and fluorescence mode on a Leica DMi8 inverted microscope (Leica) with 100X/1.32 oil objective (Leica). Phase contrast and fluorescent microscopy images were visualized and cropped using the software Fiji (68). They were segmented using the AI-based tool Omnipose (69) specifically trained with a comprehensive dataset of *C. glutamicum* images, using the phase contrast channel. Masks were manually corrected and quantitative analyses were conducted with the Fiji plugin MicrobeJ (70) to generate fluorescent intensity heat maps and profile alignments. Heat maps represent the averaged localization of the fluorescent-tagged protein on a representative cell.

## Supplementary Material

Appendix 01 (PDF)

## Data Availability

The atomic coordinates and cryo-EM map have been deposited in the Protein Data Bank (PDB) and the Electron Microscopy Data Bank (EMBD) under the accession codes 9GK2 (34) and 51414 (35) respectively. Phylogenetic analysis data have been deposited in the Mendeley Data repository (10.17632/brj488xgky.1) (71). Plasmids for the expression of PS2 (A236) or PS2^SpyTAG^ (A242) in *C. glutamicum* have been deposited at Addgene under the Addgene ID 237442 (60) and 237443 (46), respectively.

## References

[1] A. L. Houwink, A macromolecular mono-layer in the cell wall of Spirillum spec. Biochim. Biophys. Acta **10**, 360–366 (1953). 13058992 10.1016/0006-3002(53)90266-2

[2] U. B. Sleytr, B. Schuster, E. M. Egelseer, D. Pum, S-layers: Principles and applications. FEMS Microbiol. Rev. **38**, 823–864 (2014). 24483139 10.1111/1574-6976.12063 PMC4232325

[3] D. Pum, A. Breitwieser, U. B. Sleytr, Patterns in nature—S-layer lattices of bacterial and archaeal cells. Crystals **11**, 869 (2021).

[4] T. A. M. Bharat, A. von Kügelgen, V. Alva, Molecular logic of prokaryotic surface layer structures. Trends Microbiol. **29**, 405–415 (2021). 33121898 10.1016/j.tim.2020.09.009 PMC8559796

[5] T. J. Beveridge , Functions of S-layers. FEMS Microbiol. Rev. **20** (1–2), 99–149 (1997). 9276929 10.1111/j.1574-6976.1997.tb00305.x

[6] D. Alp, H. Kuleaşan, Altıntaş A. Korkut, The importance of the S-layer on the adhesion and aggregation ability of lactic acid bacteria. Mol. Biol. Rep. **47**, 3449–3457 (2020). 32279212 10.1007/s11033-020-05430-6

[7] C. Zhang , Cell structure changes in the hyperthermophilic crenarchaeon *Sulfolobus islandicus* lacking the S-layer. MBio **10**, e01589 (2019). 31455649 10.1128/mBio.01589-19 PMC6712394

[8] A. Fioravanti , Structure of S-layer protein Sap reveals a mechanism for therapeutic intervention in anthrax. Nat. Microbiol. **4**, 1805–1814 (2019). 31308522 10.1038/s41564-019-0499-1

[9] A. von Kügelgen , Membraneless channels sieve cations in ammonia-oxidizing marine archaea. Nature **630**, 230–236 (2024). 38811725 10.1038/s41586-024-07462-5 PMC11153153

[10] A. Fioravanti, M. Mathelie-Guinlet, Y. F. Dufrêne, H. Remaut, K. E. Nelson, The *Bacillus anthracis* S-layer is an exoskeleton-like structure that imparts mechanical and osmotic stabilization to the cell wall. PNAS Nexus **1**, pgac121 (2022). 36714836 10.1093/pnasnexus/pgac121 PMC9802277

[11] A. Sogues , Structure and function of the EA1 surface layer of *Bacillus anthracis*. Nat. Commun. **14**, 7051 (2023). 37923757 10.1038/s41467-023-42826-x PMC10624894

[12] R. P. Fagan, N. F. Fairweather, Biogenesis and functions of bacterial S-layers. Nat. Rev. Microbiol. **12**, 211–222 (2014). 24509785 10.1038/nrmicro3213

[13] J. A. Kirk , New class of precision antimicrobials redefines role of *Clostridium difficile* S-layer in virulence and viability. Sci. Transl. Med. **9**, eaah6813 (2017). 28878013 10.1126/scitranslmed.aah6813 PMC5603275

[14] S. Wang , Revealing roles of S-layer protein (SlpA) in *Clostridioides difficile* pathogenicity by generating the first *slpA* gene deletion mutant. Microbiol. Spectr. **12**, e04005 (2024). 38709045 10.1128/spectrum.04005-23 PMC11237437

[15] J. Y. Lee, Y. A. Na, E. Kim, H. S. Lee, P. Kim, The Actinobacterium Corynebacterium glutamicum, an Industrial Workhorse. J. Microbiol. Biotechnol. **26**, 807–822 (2016). 26838341 10.4014/jmb.1601.01053

[16] C. L. Dulberger, E. J. Rubin, C. C. Boutte, The mycobacterial cell envelope - A moving target. Nat. Rev. Microbiol. **12**, 211–222 (2019). 10.1038/s41579-019-0273-7 31728063

[17] M. Letek , DivIVA is required for polar growth in the MreB-lacking rod-shaped actinomycete corynebacterium glutamicum. J. Bacteriol. **190**, 3283–3292 (2008). 18296522 10.1128/JB.01934-07 PMC2347398

[18] E. C. Garner , Coupled circumferential motions of the cell wall synthesis machinery and MreB filaments in B. subtilis. Science **333**, 222–225 (2011). 21636745 10.1126/science.1203285 PMC3235694

[19] J. L. Peyret , Characterization of the *cspB* gene encoding Ps2, an ordered surface-layer protein in *Corynebacterium glutamicum*. Mol. Microbiol. **9**, 97–109 (1993). 8412676 10.1111/j.1365-2958.1993.tb01672.x

[20] S. Scheuring , Charting and unzipping the surface layer of Corynebacterium glutamicum with the atomic force microscope. Mol. Microbiol. **44**, 675–684 (2002). 11994150 10.1046/j.1365-2958.2002.02864.x

[21] G. Joliff , Cloning and nucleotide sequence of the *csp1* gene encoding PS1, one of the two major secreted proteins of *Corynebacterium glutamicum*: The deduced N-terminal region of PS1 is similar to the *Mycobacterium* antigen 85 complex. Mol. Microbiol. **6**, 2349–2362 (1992). 1406274 10.1111/j.1365-2958.1992.tb01410.x

[22] N. Hansmeier , Classification of hyper-variable Corynebacterium glutamicum surface-layer proteins by sequence analyses and atomic force microscopy. J. Biotechnol. **112**, 177–193 (2004). 15288952 10.1016/j.jbiotec.2004.03.020

[23] M. Chami , The S-layer protein of Corynebacterium glutamicum is anchored to the cell wall by its C-terminal hydrophobic domain. Mol. Microbiol. **23**, 483–492 (1997). 9044282 10.1046/j.1365-2958.1997.d01-1868.x

[24] N. Bayan, C. Houssin, M. Chami, G. Leblon, Mycomembrane and S-layer: Two important structures of Corynebacterium glutamicum cell envelope with promising biotechnology applications. J. Biotechnol. **104**, 55–67 (2003). 12948629 10.1016/s0168-1656(03)00163-9

[25] L. Gambelli , Architecture and modular assembly of Sulfolobus S-layers revealed by electron cryotomography. Proc. Natl. Acad. Sci. U. S. A. **116**, 25278–25286 (2019). 31767763 10.1073/pnas.1911262116 PMC6911244

[26] R. J. Blackler , Structural basis of cell wall anchoring by SLH domains in *Paenibacillus alvei*. Nat. Commun. **9**, 3120 (2018). 30087354 10.1038/s41467-018-05471-3 PMC6081394

[27] S. E. Willing , Clostridium difficile surface proteins are anchored to the cell wall using CWB2 motifs that recognise the anionic polymer PSII. Mol. Microbiol. **96**, 596–608 (2015). 25649385 10.1111/mmi.12958 PMC4973711

[28] A. von Kügelgen , In situ structure of an intact lipopolysaccharide-bound bacterial surface layer. Cell **180**, 348–358.e15 (2020). 31883796 10.1016/j.cell.2019.12.006 PMC6978808

[29] M. Herdman , Cell cycle dependent coordination of surface layer biogenesis in *Caulobacter crescentus*. Nat. Commun. **15**, 3355 (2024). 38637514 10.1038/s41467-024-47529-5 PMC11026435

[30] P. Oatley, J. A. Kirk, S. Ma, S. Jones, R. P. Fagan, Spatial organization of Clostridium difficile S-layer biogenesis. Sci. Rep. **10**, 14089 (2020). 32839524 10.1038/s41598-020-71059-x PMC7445750

[31] C. J. Comerci , Topologically-guided continuous protein crystallization controls bacterial surface layer self-assembly. Nat. Commun. **10**, 2731 (2019). 31227690 10.1038/s41467-019-10650-x PMC6588578

[32] N. Hansmeier , The surface (S)-layer gene cspB of Corynebacterium glutamicum is transcriptionally activated by a LuxR-type regulator and located on a 6 kb genomic island absent from the type strain ATCC 13032. Microbiology **152**, 923–935 (2006). 16549657 10.1099/mic.0.28673-0

[33] E. Johnston, B. Isbilir, V. Alva, T. Bharat, J. P. K. Doye, Punctuated and continuous structural diversity of S-layers across the prokaryotic tree of life. bioRxiv [Preprint] (2024). 10.1101/2024.05.28.596244 (Accessed 28 May 2024).

[34] A. Sogues, M. Sleutel, H. Remaut, Surface-layer (S-layer) PS2 protein from Corynebacterium glutamicum. Protein Data Bank. https://www.rcsb.org/structure/9GK2. Deposited 23 August 2024.

[35] A. Sogues, M. Sleutel, H. Remaut, Surface-layer (S-layer) PS2 protein from Corynebacterium glutamicum. Electron Microscopy Data Bank. https://www.ebi.ac.uk/emdb/EMD-51414. Deposited 23 August 2024.

[36] B. Isbilir, A. Yeates, V. Alva, T. Bharat, Mapping the ultrastructural topology of the corynebacterial cell surface. PLoS Biol. **23**, 4 (2025). 10.1371/journal.pbio.3003130 PMC12021427 40233127

[37] W. O. Saxton, W. Baumeister, Principles of organization in S layers. J. Mol. Biol. **187**, 251–253 (1986). 3701866 10.1016/0022-2836(86)90232-9

[38] E. Baranova , SbsB structure and lattice reconstruction unveil Ca2+ triggered S-layer assembly. Nat. Commun. **487**, 119-122 (2012). 10.1038/nature11155 22722836

[39] P. Lanzoni-Mangutchi , Structure and assembly of the S-layer in *C. difficile*. Nat. Commun. **13**, 970 (2022). 35217634 10.1038/s41467-022-28196-w PMC8881574

[40] M. Herdman , High-resolution mapping of metal ions reveals principles of surface layer assembly in Caulobacter crescentus cells. Structure **30**, 215–228.e5 (2021). 34800371 10.1016/j.str.2021.10.012 PMC8828063

[41] Y. Matsuda , Double mutation of cell wall proteins CspB and PBP1a increases secretion of the antibody Fab fragment from Corynebacterium glutamicum. Microb. Cell Fact. **13**, 56 (2014). 24731213 10.1186/1475-2859-13-56 PMC4021378

[42] J. A. Kirk , New class of precision antimicrobials redefines role of *Clostridium difficile* s-layer in virulence and viability. Sci. Transl. Med. **9**, eaah6813 (2017). 28878013 10.1126/scitranslmed.aah6813 PMC5603275

[43] D. Zhang , Physiological changes and growth behavior of *Corynebacterium glutamicum* cells in biofilm. Front. Microbiol. **13**, 983545 (2022). 36110303 10.3389/fmicb.2022.983545 PMC9468548

[44] B. Zakeri , Peptide tag forming a rapid covalent bond to a protein, through engineering a bacterial adhesin. Proc. Natl. Acad. Sci. U. S. A. **109**, E690 (2012). 22366317 10.1073/pnas.1115485109 PMC3311370

[45] P. Ravasi, S. Peiru, H. Gramajo, H. G. Menzella, Design and testing of a synthetic biology framework for genetic engineering of *Corynebacterium glutamicum*. Microb. Cell Fact. **11**, 147 (2012). 23134565 10.1186/1475-2859-11-147 PMC3539996

[46] A. Sogues, H. Remaut, pTGR-PS2-SpyTAG: plasmid expressing PS2 (SpyTAG) S-layer from C. glutamicum under its native promoter. Addgene. https://www.addgene.org/237443/. Deposited 31 March 2025.

[47] M. Farid. Abdul-Halim , Lipid anchoring of Archaeosortase substrates and midcell growth in Haloarchaea. MBio **11**, e00349 (2020). 32209681 10.1128/mBio.00349-20 PMC7157517

[48] F. M. Meyer, M. Bramkamp, Cell wall synthesizing complexes in Mycobacteriales. Curr. Opin. Microbiol. **79**, 102478 (2024). 38653035 10.1016/j.mib.2024.102478

[49] A. Sogues , Essential dynamic interdependence of FtsZ and SepF for Z-ring and septum formation in Corynebacterium glutamicum. Nat. Commun. **11**, 1641 (2020). 32242019 10.1038/s41467-020-15490-8 PMC7118173

[50] T. A. M. Bharat , Structure of the hexagonal surface layer on Caulobacter crescentus cells. Nat. Microbiol. **2**, 1–6 (2017). 10.1038/nmicrobiol.2017.59 PMC5699643 28418382

[51] A. von Kügelgen , Interdigitated immunoglobulin arrays form the hyperstable surface layer of the extremophilic bacterium Deinococcus radiodurans. Proc. Natl. Acad. Sci. U. S. A. **120**, e2215808120 (2023). 37043530 10.1073/pnas.2215808120 PMC10120038

[52] T. Hirasawa, M. Wachi, K. Nagai, A mutation in the *Corynebacterium glutamicum* ltsA gene causes susceptibility to lysozyme, temperature-sensitive growth, and L-glutamate production. J. Bacteriol. **182**, 2696–2701 (2000). 10781535 10.1128/jb.182.10.2696-2701.2000 PMC101969

[53] S. V. Albers, B. H. Meyer, The archaeal cell envelope. Nat. Rev. Microbiol. **9**, 414–426 (2011). 21572458 10.1038/nrmicro2576

[54] E. Soual-Hoebeke , S-layer protein production by Corynebacterium strains is dependent on the carbon source. Microbiology **145**, 3399–3408 (1999). 10627038 10.1099/00221287-145-12-3399

[55] V. M. Marando , Biosynthetic glycan labeling. J. Am. Chem. Soc. **143**, 16337–16342 (2021). 34606245 10.1021/jacs.1c07430 PMC8943913

[56] L. Thouvenel, J. Rech, C. Guilhot, J. Y. Bouet, C. Chalut, In vivo imaging of MmpL transporters reveals distinct subcellular locations for export of mycolic acids and non-essential trehalose polyphleates in the mycobacterial outer membrane. Sci. Rep. **13**, 7045 (2023). 37120636 10.1038/s41598-023-34315-4 PMC10148836

[57] X. Zhou , Sequential assembly of the septal cell envelope prior to V snapping in Corynebacterium glutamicum. Nat. Chem. Biol. **647**, 1 (2019). 10.1038/s41589-018-0206-1 PMC6693342 30664686

[58] C. Houssin, D. T. Nguyen, G. Leblon, N. Bayan, S-layer protein transport across the cell wall of Corynebacterium glutamicum: In vivo kinetics and energy requirements. FEMS Microbiol. Lett. **217**, 71–79 (2002). 12445648 10.1111/j.1574-6968.2002.tb11458.x

[59] B. R. Johnson , Conserved S-layer-associated proteins revealed by exoproteomic survey of S-layer-forming lactobacilli. Appl. Environ. Microbiol. **82**, 134–145 (2015). 26475115 10.1128/AEM.01968-15 PMC4702614

[60] A. Sogues, H. Remaut, pTGR-PS2: plasmid expressing PS2 S-layer from C. glutamicum under its native promoter. Addgene. https://www.addgene.org/237442/. Deposited 31 March 2025.

[61] A. Punjani, J. L. Rubinstein, D. J. Fleet, M. A. Brubaker, CryoSPARC: Algorithms for rapid unsupervised cryo-EM structure determination. Nat. Methods **14**, 290–296 (2017). 28165473 10.1038/nmeth.4169

[62] J. He, T. Li, S. Y. Huang, Improvement of cryo-EM maps by simultaneous local and non-local deep learning. Nat. Commun. **14**, 1–16 (2023). 37270635 10.1038/s41467-023-39031-1 PMC10239474

[63] K. Jamali , Automated model building and protein identification in cryo-EM maps. Nature **628**, 450–457 (2024). 38408488 10.1038/s41586-024-07215-4 PMC11006616

[64] J. Jumper , Highly accurate protein structure prediction with AlphaFold. Nature **596**, 583–589 (2021). 34265844 10.1038/s41586-021-03819-2 PMC8371605

[65] P. Emsley, K. Cowtan, Coot: Model-building tools for molecular graphics. Acta Crystallogr. D Biol. Crystallogr. **60**, 2126–2132 (2004). 15572765 10.1107/S0907444904019158

[66] D. Liebschner , Macromolecular structure determination using X-rays, neutrons and electrons: Recent developments in Phenix. Acta Crystallogr. D Biol Crystallogr. **75**, 861–877 (2019). 10.1107/S2059798319011471 PMC6778852 31588918

[67] E. F. Pettersen , UCSF chimeraX: Structure visualization for researchers, educators, and developers. Protein Sci. **30**, 70 (2021). 32881101 10.1002/pro.3943 PMC7737788

[68] J. Schindelin , Fiji: An open-source platform for biological-image analysis. Nat. Methods **9**, 676–682 (2012). 22743772 10.1038/nmeth.2019 PMC3855844

[69] K. J. Cutler , Omnipose: A high-precision morphology-independent solution for bacterial cell segmentation. Nat. Methods **19**, 1438–1448 (2022). 36253643 10.1038/s41592-022-01639-4 PMC9636021

[70] A. Ducret, E. M. Quardokus, Y. V. Brun, MicrobeJ, a tool for high throughput bacterial cell detection and quantitative analysis. Nat. Microbiol. **1**, 16077 (2016). 27572972 10.1038/nmicrobiol.2016.77 PMC5010025

[71] A. Sogues , Cryo-EM structure and polar assembly of the PS2 S-layer of Corynebacterium glutamicum. Mendeley Data. https://data.mendeley.com/datasets/brj488xgky/1. Deposited 18 March 2025. 10.1073/pnas.2426928122 PMC12337289 40729392

