# Supplementary material for: Cryo-EM structure and polar assembly of the PS2 S-layer of Corynebacterium glutamicum
Source: Proc Natl Acad Sci U S A. 2025 Jul 29;122(31):e2426928122. doi: 10.1073/pnas.2426928122 (PMC12337289; doi:10.1073/pnas.2426928122)
Supplement: Supplementary file 1 — Appendix 01 (PDF) [file pnas.2426928122.sapp.pdf]

Supporting Information

Ex-vivo cryo-EM structure and polar assembly of the PS2 S-layer of *Corynebacterium glutamicum*

Authors:

Adrià Sogues[1,2], Mike Sleutel[1,2], Julienne Petit[3,4], Daniela Megrian[5], Nicolas Bayan[6], Anne Marie Wehenkel[3] & Han Remaut[1,2]

Affiliations:

1. Structural and Molecular Microbiology, VIB-VUB Center for Structural Biology, VIB, Pleinlaan 2, 1050 Brussels, Belgium.
2. Structural Biology Brussels, Vrije Universiteit Brussel, VUB, Pleinlaan 2, 1050 Brussels, Belgium.
3. Institut Pasteur, Université Paris Cité, CNRS UMR 3528, Bacterial Cell Cycle Mechanisms Unit, F-75015 Paris, France.
4. Institut Pasteur, Université Paris Cité, CNRS UMR 3528, Structural Microbiology Unit, F-75015 Paris, France.
5. Bioinformatics Unit, Institut Pasteur de Montevideo, 11200 Montevideo, Uruguay.
6. Université Paris-Saclay, CEA, CNRS, Institute for Integrative Biology of the Cell (I2BC), Gif-sur-Yvette, France.

This file includes:

Figures S1-S13

Table S1-S3

SI Methods

SI References

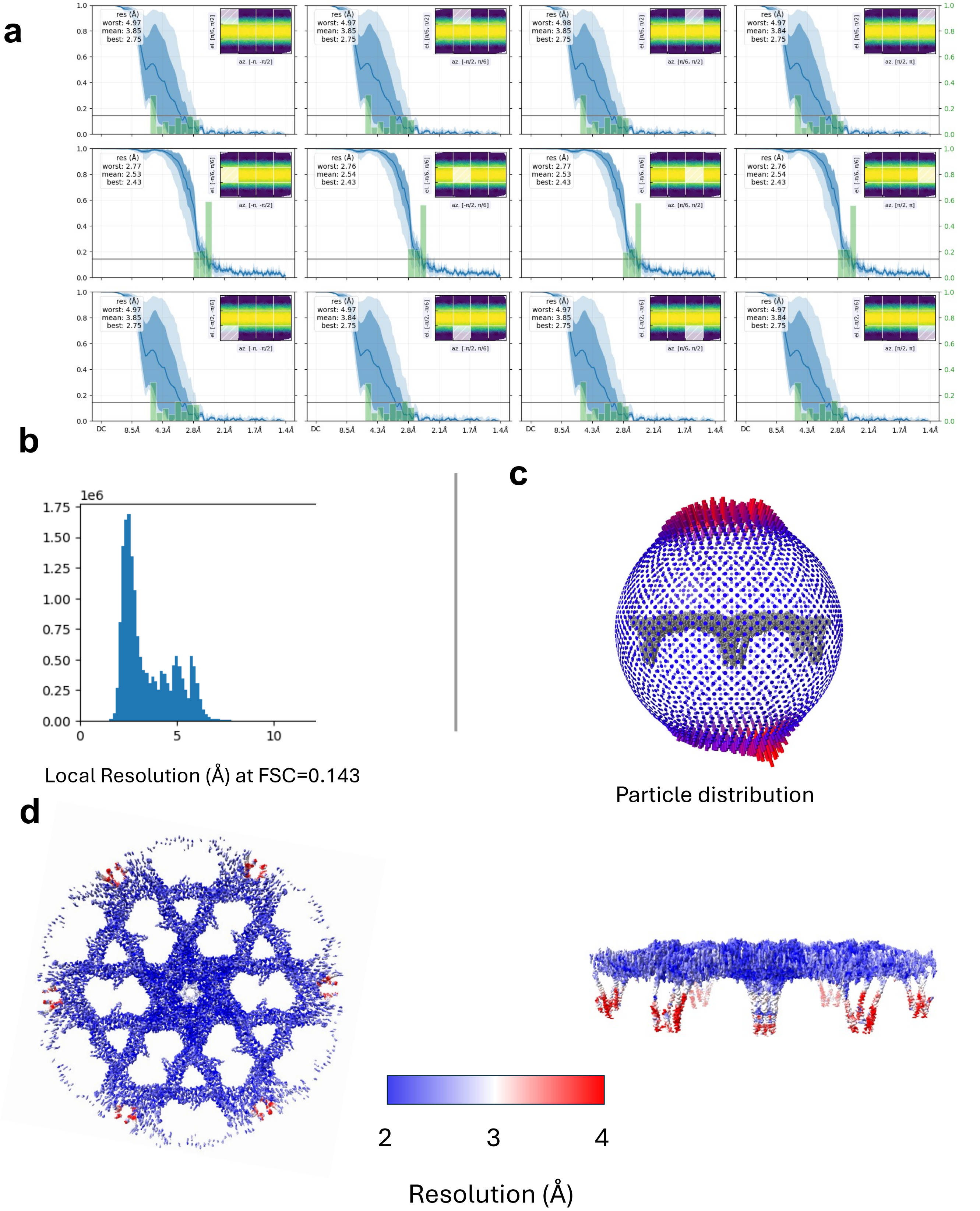

Figure S1. Single particle analysis of *ex-vivo* PS2 sheets. a. FSC variation as a function of viewing direction obtained by the orientation diagnostics jobs (CryoSPARC) shows different map resolutions. **b.** Histogram of local resolutions in voxels of the cryo-EM map (C6-symmetrised). **c.** Particle distribution mapped on the cryo-EM density map. Red indicated hiher number of particles on that orientation, an enrichment of view at 15 and 30 degrees as a result of the tilted collection. **d.** Local resolution of the C2 cryo-EM map estimated in CryoSPARC, plotted into the density, shown from the top and side

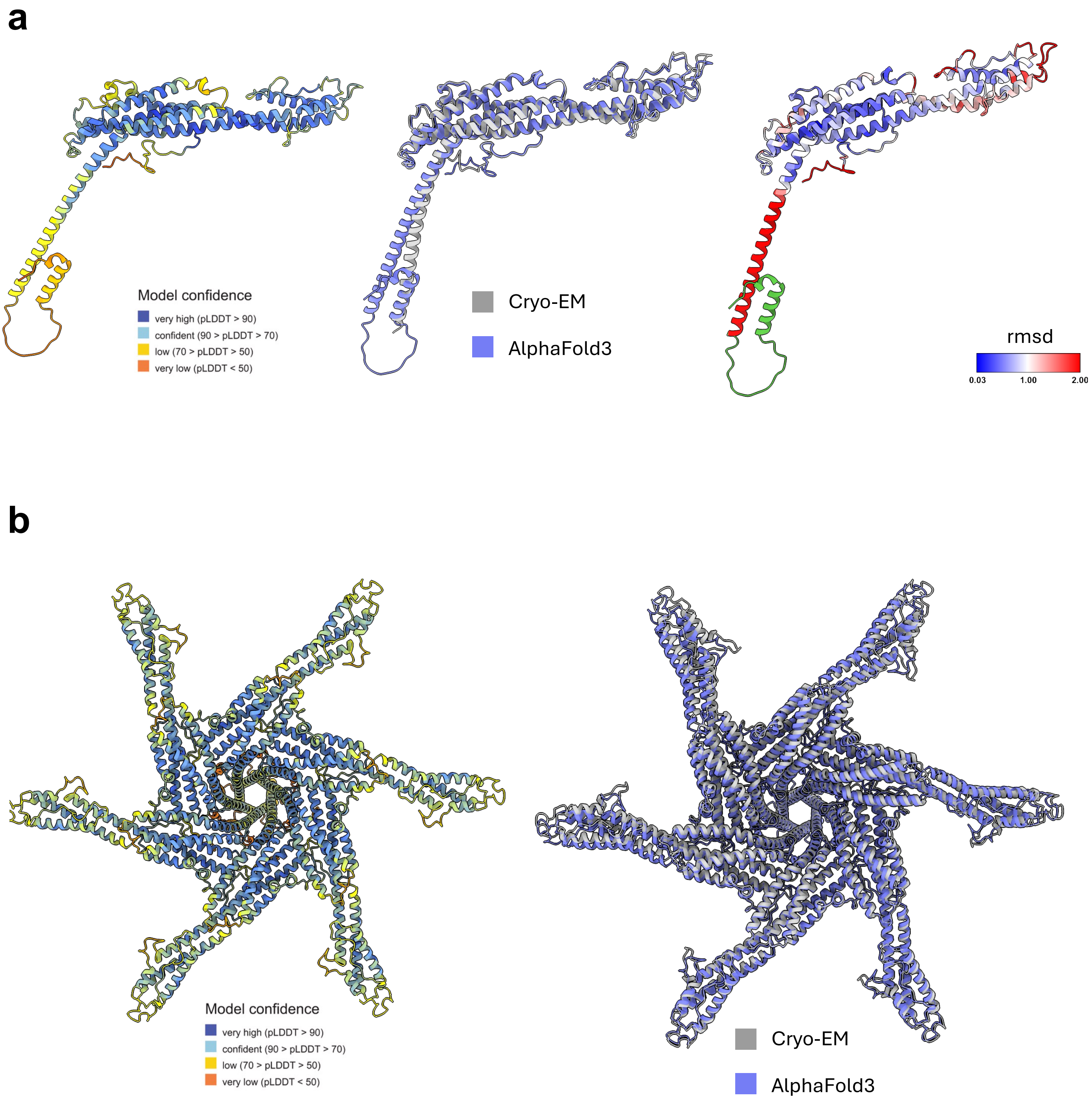

Figure S2. Prediction of the PS2 structure by AlphaFold3 compared with the Cryo-EM structure. a Left. Ribbon representation of the PS2 monomer predicted by AlphaFold3, coloured by pLDDT values. **Middle.** Superposition of the predicted PS2 monomer (purple) with the Cryo-EM determined structure (grey) RMSD 3.28 Å. **Right.** AlphaFold3 model coloured by RMSD values when superimposed onto the Cryo-EM structure. Blue indicates close matches (low RMSD values), red indicates higher RMSD deviations, and green highlights areas lacking correspondence between the two structures. **b**. **Left**. Ribbon representation of the PS2 hexamer predicted by AF3 and coloured according to pLDDT values. **Right**. Structural superposition of the predicted AF3 structure with the experimentally obtained using cryo-EM. RMSD 2.76 Å.

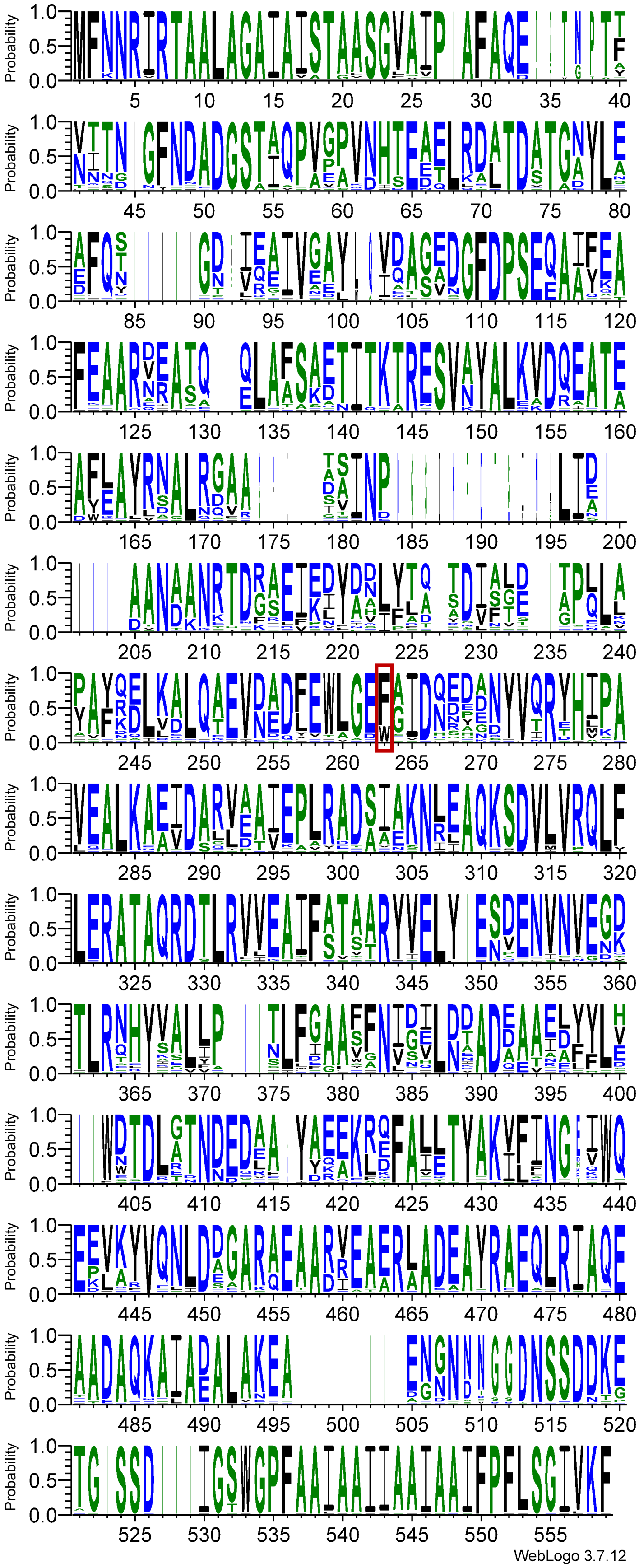

Figure S3. WebLogo of PS2. Sequence alignment obtained from the ConSurf[1] server was used as input to generate the weblogo using WebLogo3[2]. Red boxed residue indicated the distal F from the arm region.

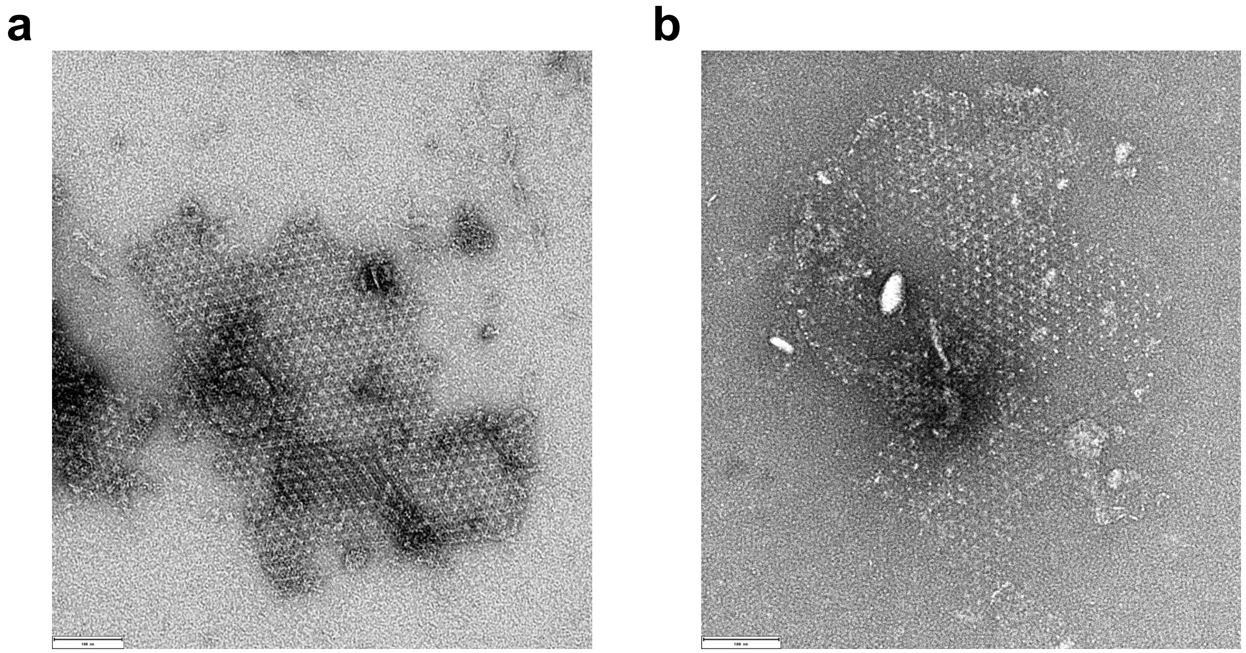

Figure S4. $PS2^{AD}$ S-layer assembly and stability are independent of divalent ions. a. NS-EM of PS2 S-layer incubated with 10 mM EDTA for a week shows the presence of assembled S-layer. **b**. NS-EM of refolded PS2 in the presence of 10 mM EDTA. Scale bar 100 nm.

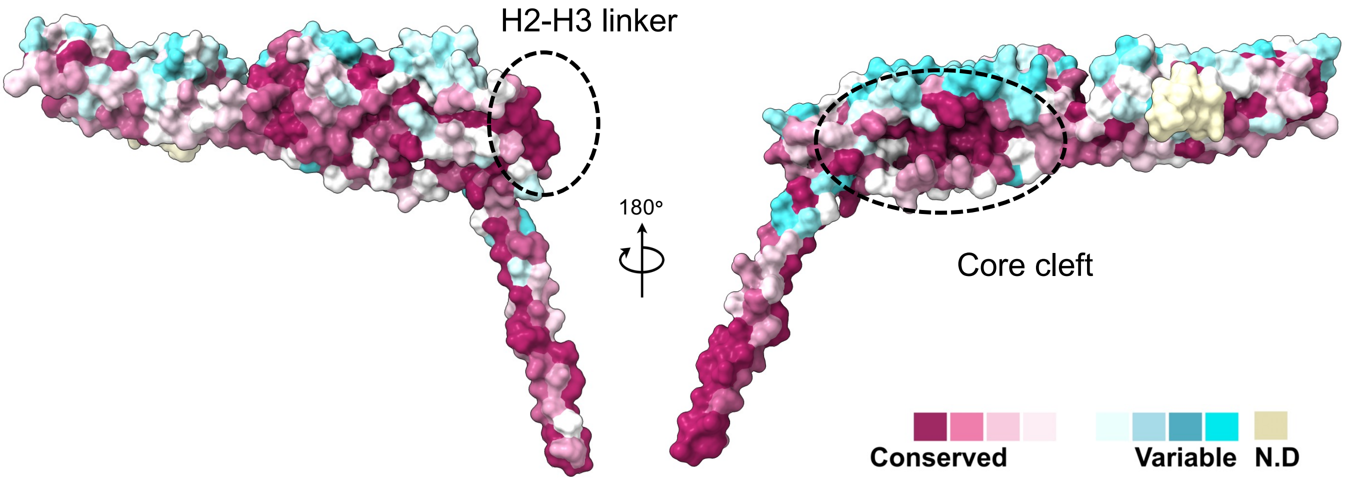

Figure S5. Conservation of the hexametric C6 interface. The conservation of the PS2 S-layer has been mapped on the structure's surface using the ConSurf server[1]. Dark pink indicates high levels of conservation. Regions involve in the hexametric interface are indicated.

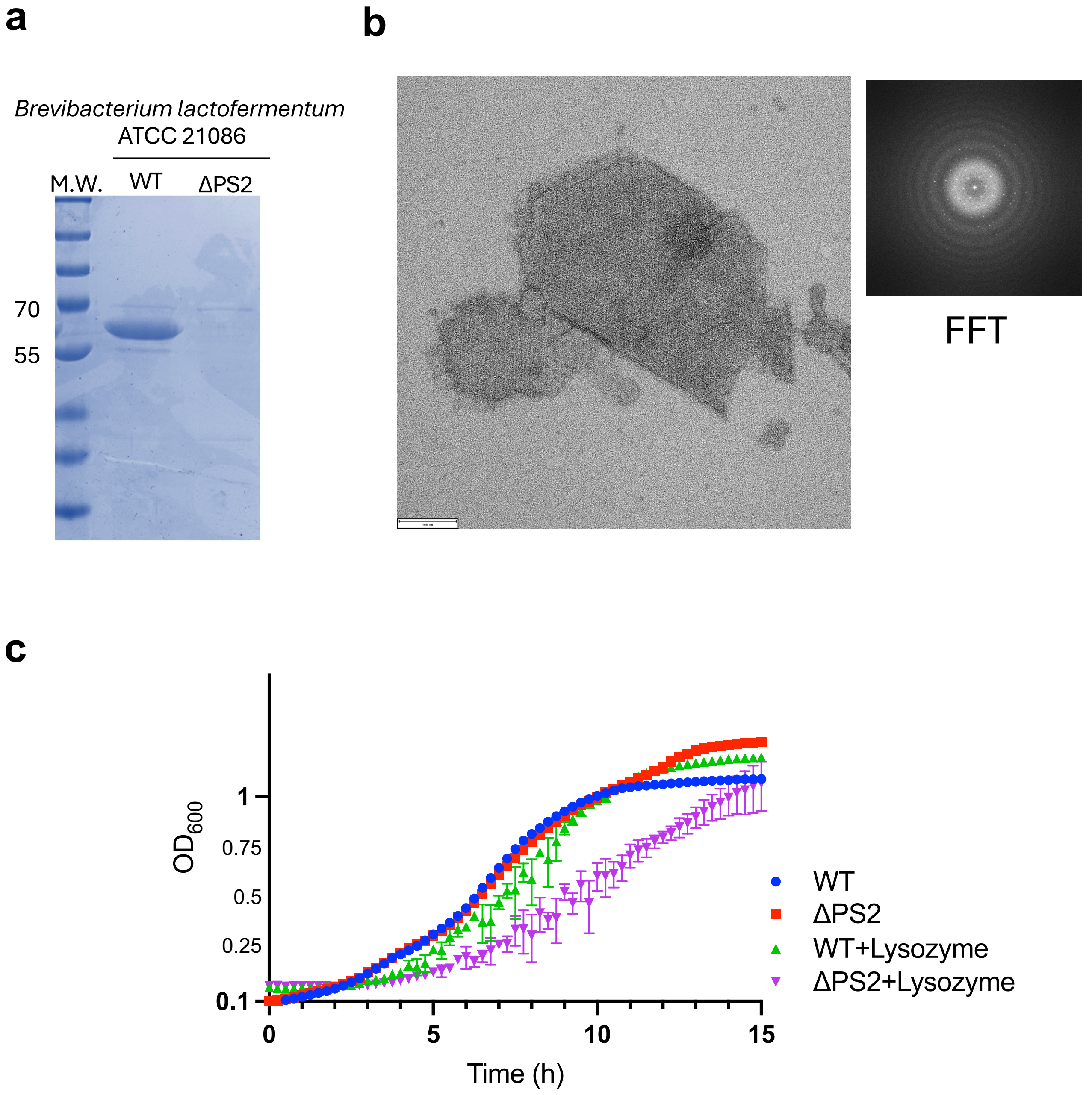

Figure S6. Confirmation of PS2 expression in the ATCC21086 strain. a. SDS-PAGE (Coomassie-stained) of SDS-extracted cell surface proteins. **b.** (left) Ex vivo purified fragment of PS2. Scale bas is 100 nm. (right) Fourier Transform of the S-layer fragment. The estimated unit cell parameters are α = β = 172 Å and γ = 60°. **c**. Growth curves of the ATCC21086 WT and ΔPS2 in LB (red and blue) or LB supplemented with 100 µg/ml of lysozyme (purple and green). Growth curve data are sample mean ± s.d., representative of n = 3 biological experiments. Lysozyme was added at the start of the measurements

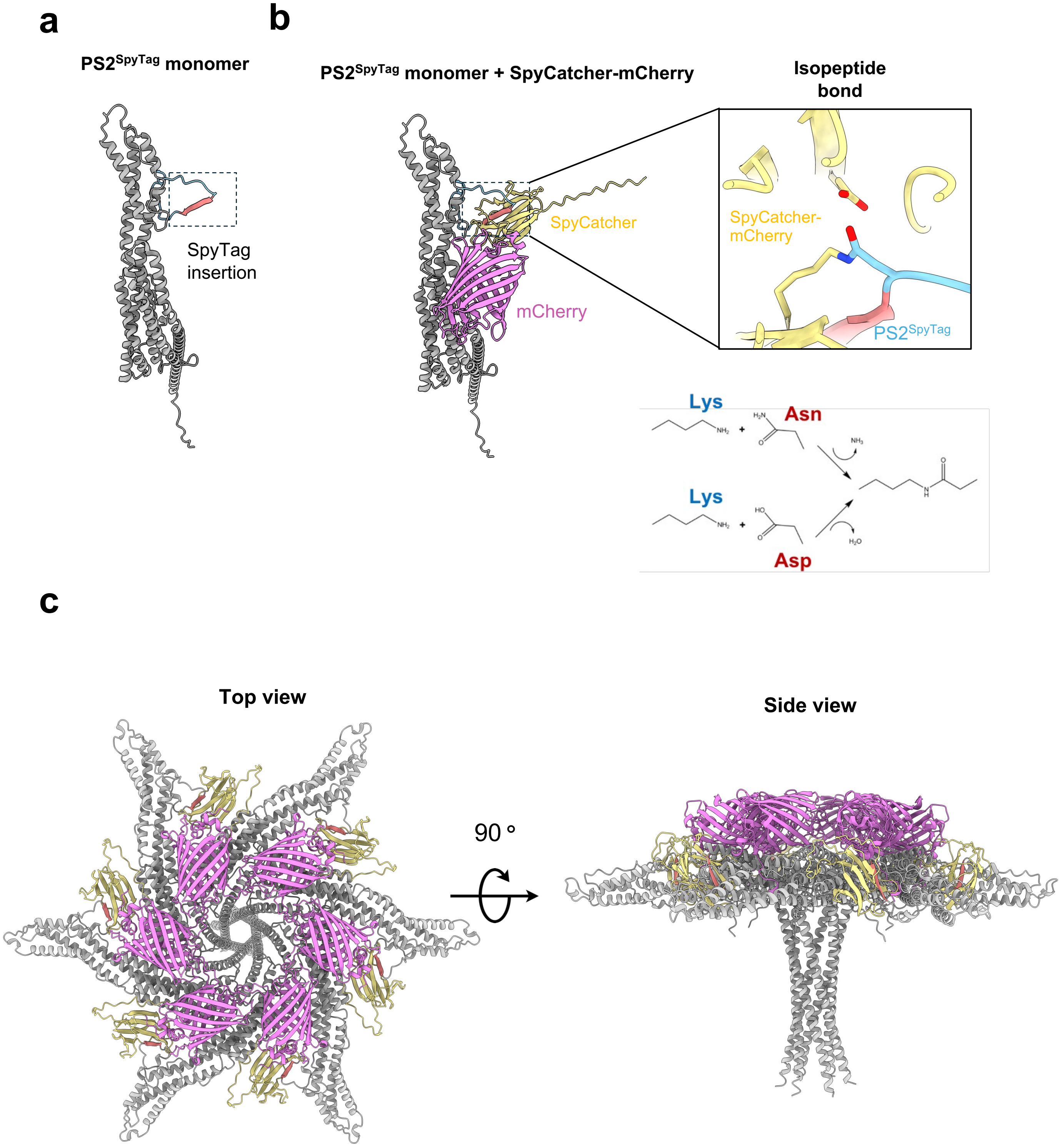

Figure S7. PS2^{SpyTag} system. a. AlphaFold3 model of the SpyTag added in the H3 insertion loop (left panel). **b.** Upon the exogenous addition of purified SpyCatcher:mCherry (middle panel) , they form an isopeptide bond (covalent bond) catalysed by a glutamic acid located in the proximity (right panel). Reaction scheme of the isopeptide bond formation (bottom panel) **c.** AlphaFold3 prediction of the PS2 hexamer with six SpyCatcher-mCherry molecules.

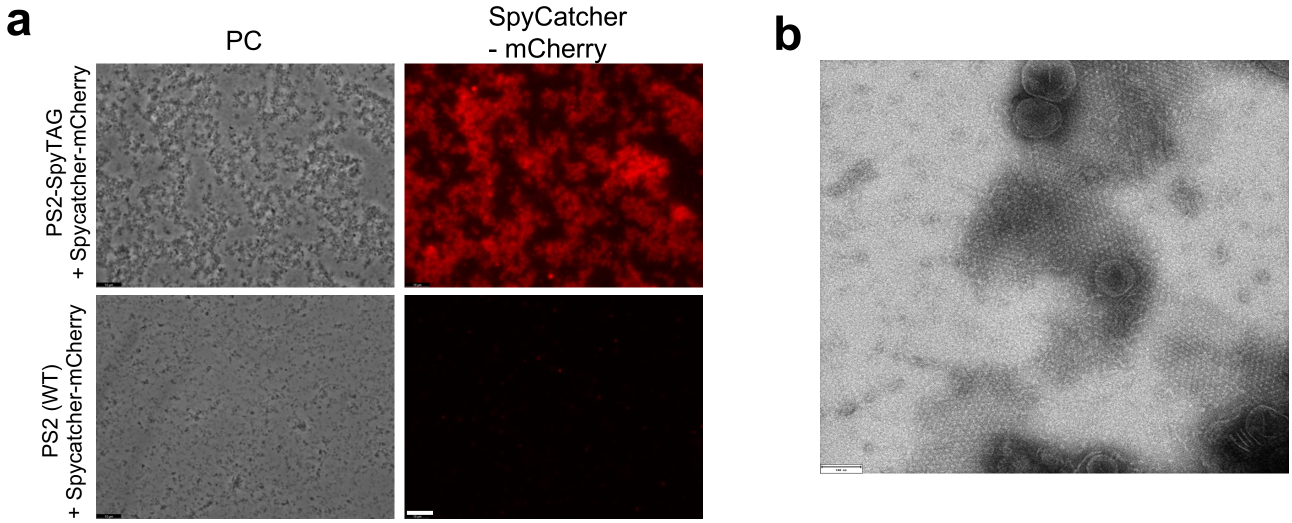

Figure S8. *In vitro* engineering of PS2. a. *E. coli* produced **r**ecombinant PS2-SpyTAG assembly domain is able to specifically bind SpyCatcher-mCherry (top) whereas the WT version shows no signal (bottom). Scale bar is 10 μM. **b**. PS2-SpyTag assembly domain still forms S-layer in vitro after being loaded with SpyCatcher-mCherry as observed using ns-EM. Scale bar is 100 nM.

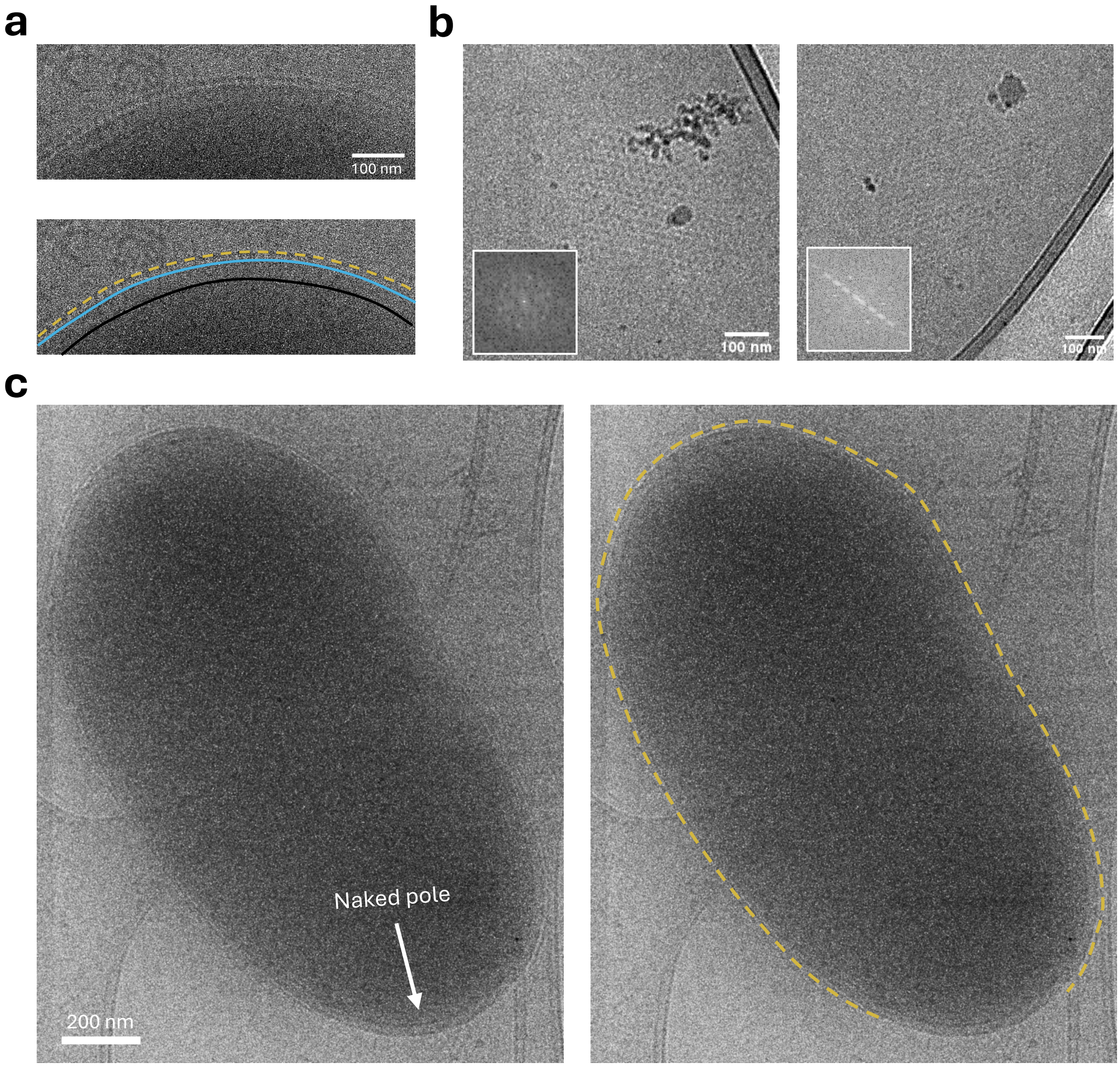

Figure S9. CryoEM imaging of the *C. glutamicum* cell envelope. (a) Close-up cryoEM image of intact *C. glutamicum* ATCC13032-PS2-SpyTAG cell showing the PS2 S-layer (dotted yellow trace line), mycomembrane (blue trace line) and cytoplasmic membrane (black trace line). **(b)** CryoEM images of *C. glutamicum* ATCC13032-PS2-SpyTAG cells show the presence of shed S-layer fragments (powerspectrum inset). **(c)** cryoEM image of *C. glutamicum* ATCC13032-PS2-SpyTAG cell with indication of the PS2 S-layer (yellow dotted line). The cell shows the presence of the S-layer throughout the cell surface with the exception of a single pole, likely the new pole, shortly after cell division (i.e. see Figure 5 for pulse chase labelling experiments)

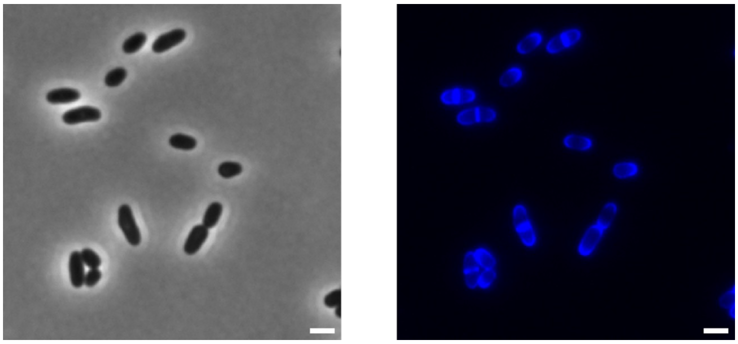

Figure S10. HADA labelling of *C. glutamicum* ATCC13032. HADA is a fluorescent D-amino acid used to label newly inserted peptidoglycan. In *C. glutamicum*, peptidoglycan is synthesized at the poles and septum. Scale bar is 2 μM.

a

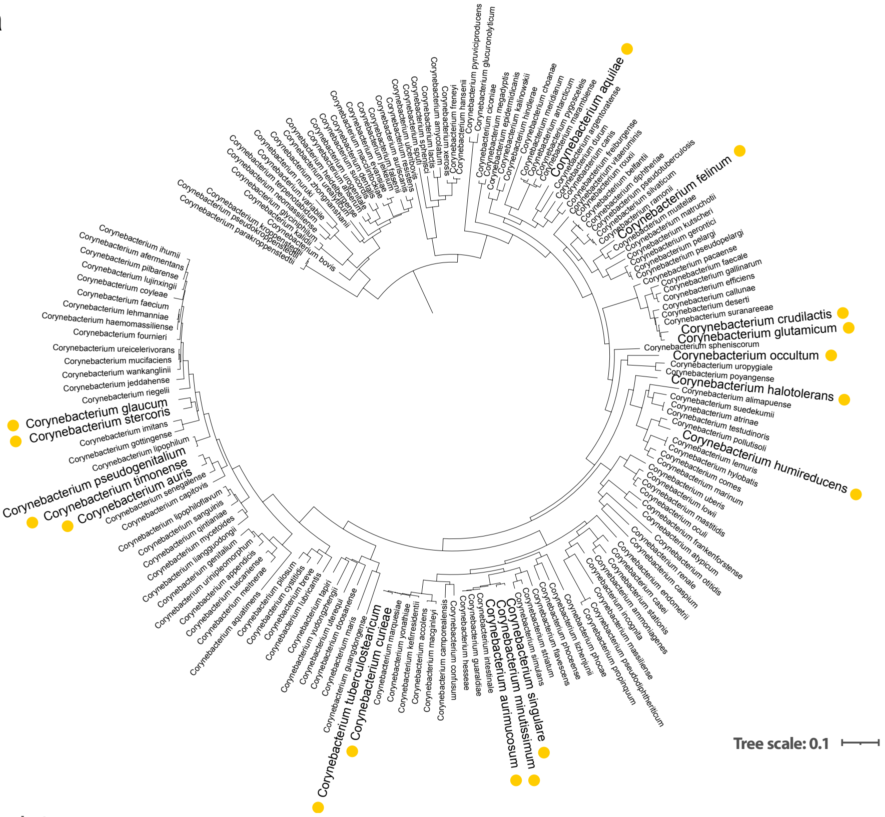

Corynebacterium aquilae
Corynebacterium felinum
Corynebacterium crudilactis
Corynebacterium glutamicum
Corynebacterium occultum
Corynebacterium halotolerans
Corynebacterium humireducens
Corynebacterium singulare
Corynebacterium minutissimum
Corynebacterium aurimucosum
Corynebacterium tuberculostearicum
Corynebacterium curieae
Corynebacterium auris
Corynebacterium timonense
Corynebacterium pseudogenitalium
Corynebacterium stercoris
Corynebacterium glaucum
Tree scale: 0.1

b

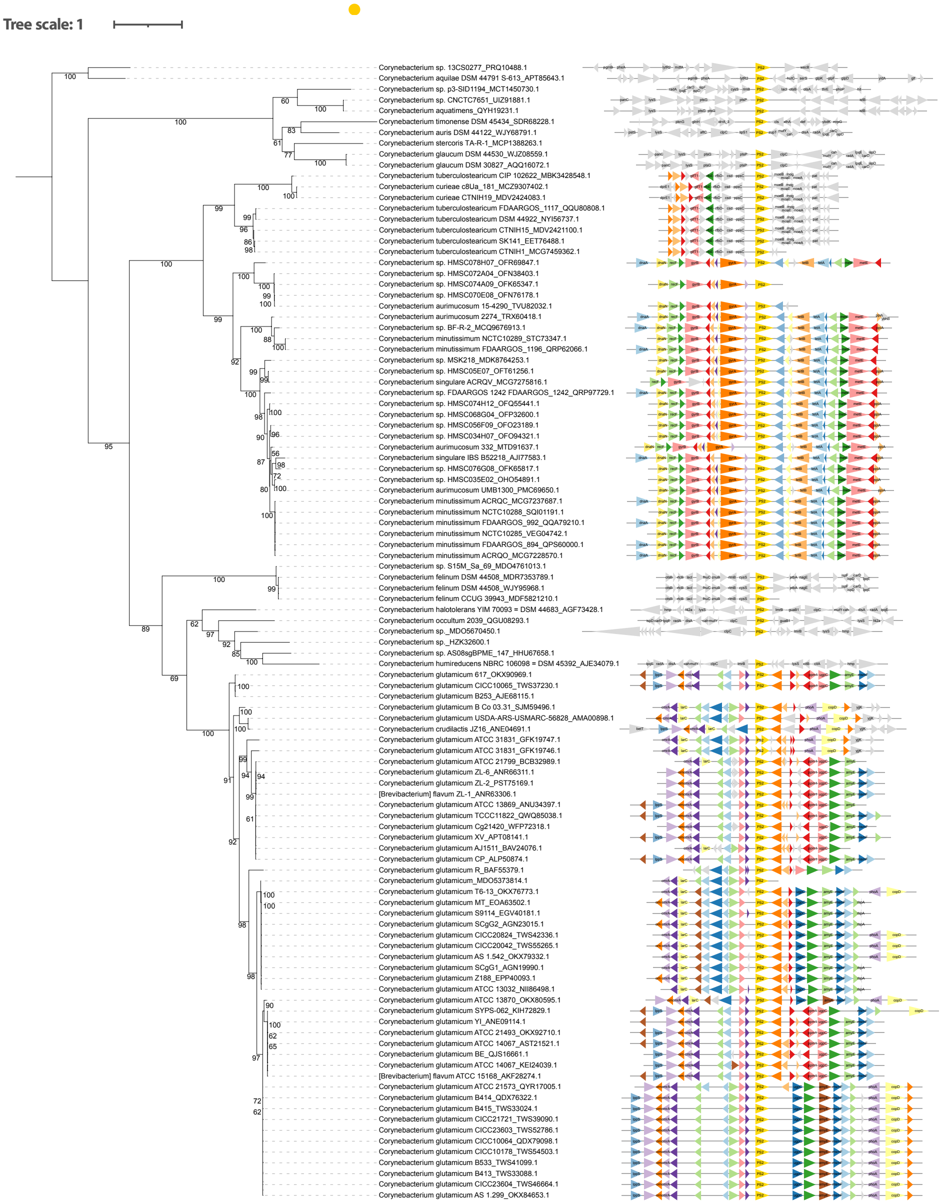

Tree scale: 1

Figure S11. PS2 phylogeny and genome context show multiple recombination events. a. Phyletic pattern of the presence of PS2 in a reference phylogeny of the *Corynebacterium* genus. Yellow circles indicate that at least one strain for that species codes for protein PS2. **b**. Phylogenetic tree of protein PS2 (centred in yellow) and genomic context. Triangles in colours correspond to genes frequently found in the same locus as *cspB* (PS2). Triangles without labels correspond to genes of unknown function. Numbers on branches correspond to ultrafast bootstrap supports.

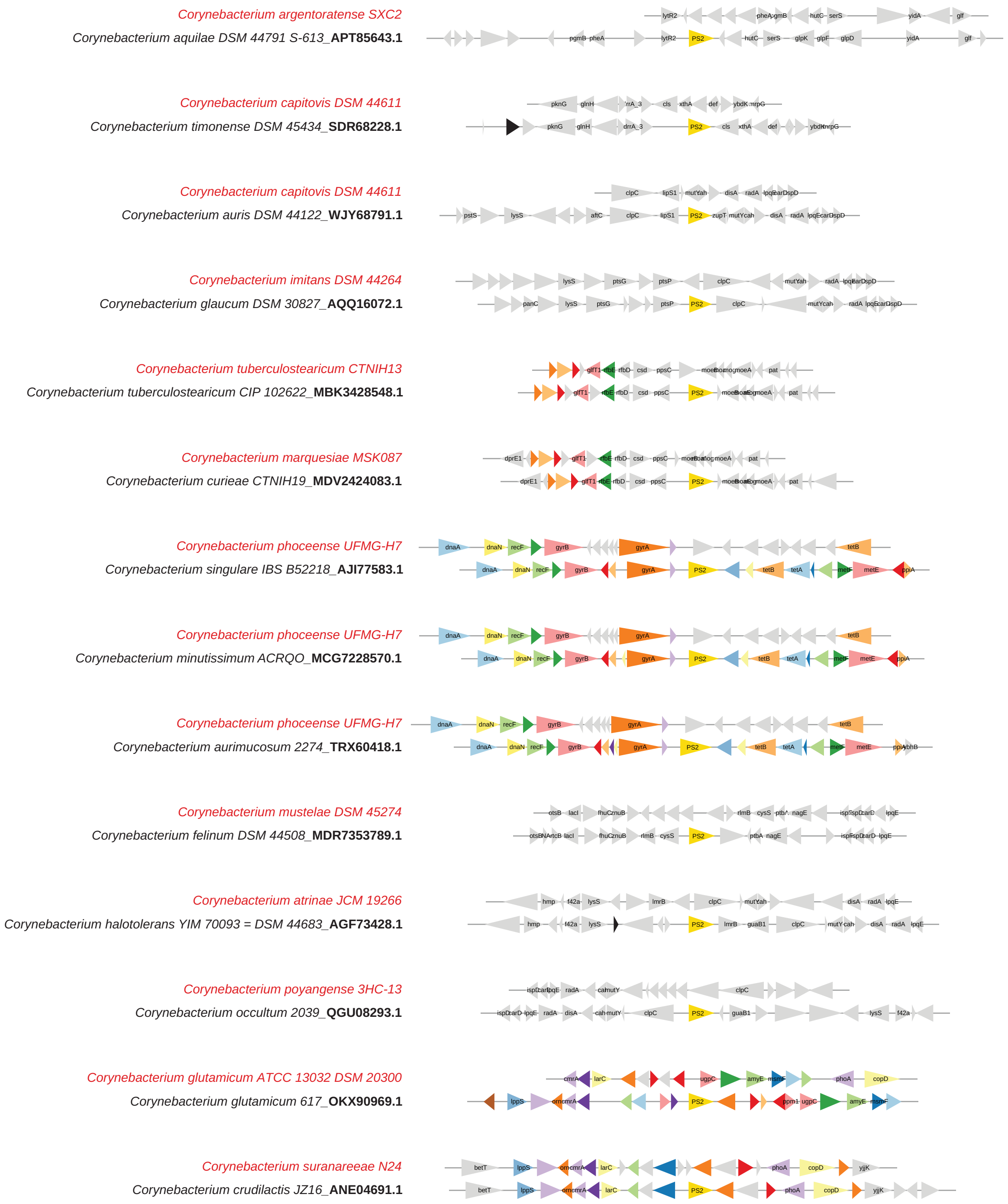

Figure S12. Comparison of the genomic context of strains that code for PS2 and closely related strains that do not code for PS2. Triangles in colors correspond to genes frequently found in the same locus as *cspB* (PS2). Triangles without labels correspond to genes of unknown function.

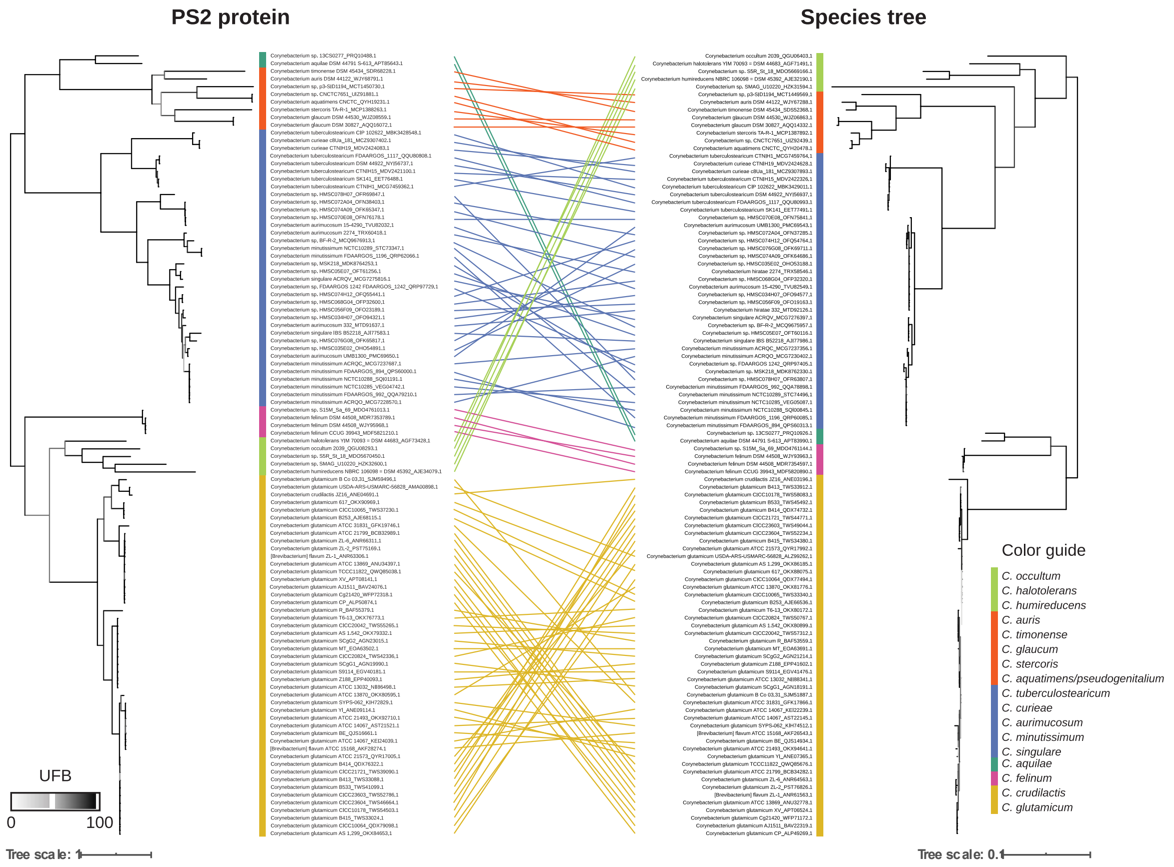

Figure S13. Correspondence between the PS2 protein phylogeny and the species tree of the Corynebacterium genus. The species tree includes only the strains that contain protein PS2. The colors group species and strains that are monophyletic in the full Corynebacterium genus species phylogeny. The branches are colored in shades of gray according to the ultrafast bootstrap (UFB) supports.

Oligo number	Name	Sequence (5' --> 3')
**p849**	F_PS2	GGAGATATACAAATGCaggaaaccactgtaaccaccaacgg
**p868**	R_PS2_FL_V2	CAGGTCAAGCTTAttaGTGATGATGATGGTGATGGAACTTAACGATACCGGAGAGGAATG
**p850**	R_PS2	CAGGTCAAGCTTAttaGTGATGATGATGGTGATGCCAGGATCCGATGTCAGAAGAACCG
**p321**	F_open_pASK_3	TAATAAGCTTGACCTGTGAAG
**p322**	R_open_pASK_3	CATTTGTATATCTCCTTCTTAAAG
**p873**	F_Ps2_spyTAG_V3	ATCGTCATGGTAGACGCCTACAAACGCTACAAAGGTTCTATCAACCCAGATACCTCTATCA ACCTACTGAT
**p874**	R_Ps2_spyTAG_V3	CGTCTACCATGACGATGTGCGGCACGCCCCGACCGCCATCTGGGTTGATAGAGATAGC TGCATCG
**p862**	F_pTGR5_open	TAAAGCGGCCGCTTAAG
**p863**	R_pTGR5_open	AATATGCGGCCGCATATAT

Plasmid name	Vector family	Inducer	Resistance	Expressing gene	Reference
A231	pASK-IBA3plus	anhydrotetracycline	Ampi	cspB assebly domain	This study
A232	pASK-IBA3plus	anhydrotetracycline	Ampi	cspB full length	This study
A233	pASK-IBA3plus	anhydrotetracycline	Ampi	spycatcher:sfGFP	This study
A234	pASK-IBA3plus	anhydrotetracycline	Ampi	spycatcher:mCherry	This study
A235	pASK-IBA3plus	anhydrotetracycline	Ampi	cspB assebly domain-SpyTag	This study
A236	pTGR5	N/A (cspB native promoter)	Kana	cspB full length	This study
A242	pTGR5	N/A (cspB native promoter)	Kana	cspB full length SpyTAG	This study

Strains	Characteristics	Source
E. coli		
DH5α	F- endA1 Φ80dlacZΔM15 Δ(lacZYA-argF)U169 recA1 relA1 hsdR17(rK–mK+) deoR supE44 thi-1 gyrA96 phoA λ–; strain used for general cloning procedures	NEB (#C2987H)
BL21 (DE3)	F- ompT hsdSB(rB–mB–) gal dcm (DE3); host for protein production	NEB (#C2527H)
C. glutamicum		
ATCC13032	Biotin-auxotrophic wild type	BCCM(#LMG 19741)
ATCC13032 *icd::cspB*	Biotin-auxotrophic wild type. Insertion of the cspB gene in the icd (isocitrate deshydrogenase) locus	Nicolas Bayan lab
ATCC13032 -PS2-SpyTAG	Biotin-auxotrophic wild type transformed with plasmid A242. Kana resistance.	This study
Brevibacterium lactofermentum ATCC 21086	Biotin-auxotrophic and restriction-deficient wild-type	Peyret JL, et al. 1993 [3]
Brevibacterium lactofermentum ATCC 21086 Δ *cspB*	Biotin-auxotrophic and restriction-deficientΔ *cspB*	Peyret JL, et al. 1993 [3]

Table S1. The top table lists the oligonucleotides, the middle table lists the plasmids, and the bottom table lists the strains used in this study.

Name	Protein Sequence
PS2 Full length	MFNNRIRTAALAGAIAISTAASGVAIPAFAQETNPTFNITNGFNDADGSTIQPVGPVNHTEETLRDLTDSTGAYLEEFQNGTVEEIVEAYLQVQASADGFDPSEQAAYEAFEAARVRASQELAASAETITKTRESVAYALKVDQEATAAFEAYRNALRDAAISINPDGSINPDTSINLLIDAANAANRTDRAEIEDYAHLYTQTDIALETPQLAYAFQDLKALQAEVDADFEWLGEFGIDQEDGNYVQRYHLPAVEALKAEVDARVAAIEPLRADSIAKNLEAQKSDVLVRQLFLERATAQRDTLRVVEAIFSTSARYVELYENVENVNVENKTLRQHYSALIPNLFIAAVANISELNAADAEAAAYYLHWDTDLATNDEDEAYYKAKLDFAIETYAKILFNGEVWQEPLAYVQNLDAGARQEAADREAARAADEAYRAEQLRIAQEAADAQKAIAEALAKEAEGNNDNSSDNTETGSSDIGSWGPFAAIAAIIAAIAAIFPFLSGIVKF
PS2 Assembly domain	MQETNPTFNITNGFNDADGSTIQPVGPVNHTEETLRDLTDSTGAYLEEFQNGTVEEIVEAYLQVQASADGFDPSEQAAYEAFEAARVRASQELAASAETITKTRESVAYALKVDQEATAAFEAYRNALRDAAISINPDGSINPDTSINLLIDAANAANRTDRAEIEDYAHLYTQTDIALETPQLAYAFQDLKALQAEVDADFEWLGEFGIDQEDGNYVQRYHLPAVEALKAEVDARVAAIEPLRADSIAKNLEAQKSDVLVRQLFLERATAQRDTLRVVEAIFSTSARYVELYENVENVNVENKTLRQHYSALIPNLFIAAVANISELNAADAEAAAYYLHWDTDLATNDEDEAYYKAKLDFAIETYAKILFNGEVWQEPLAYVQNLDAGARQEAADREAARAADEAYRAEQLRIAQEAADAQKAIAEALAKEAEGNNDNSSDNTETGSSDIGSW
PS2 signal peptide	MFNNRIRTAALAGAIAISTAASGVAIPAFA
PS2 SpyTag	MFNNRIRTAALAGAIAISTAASGVAIPAFAQETNPTFNITNGFNDADGSTIQPVGPVNHTEETLRDLTDSTGAYLEEFQNGTVEEIVEAYLQVQASADGFDPSEQAAYEAFEAARVRASQELAASAETITKTRESVAYALKVDQEATAAFEAYRNALRDAAISINPDGGRGVPHIVMVDAYKRYKGSINPDTSINLLIDAANAANRTDRAEIEDYAHLYTQTDIALETPQLAYAFQDLKALQAEVDADFEWLGEFGIDQEDGNYVQRYHLPAVEALKAEVDARVAAIEPLRADSIAKNLEAQKSDVLVRQLFLERATAQRDTLRVVEAIFSTSARYVELYENVENVNVENKTLRQHYSALIPNLFIAAVANISELNAADAEAAAYYLHWDTDLATNDEDEAYYKAKLDFAIETYAKILFNGEVWQEPLAYVQNLDAGARQEAADREAARAADEAYRAEQLRIAQEAADAQKAIAEALAKEAEGNNDNSSDNTETGSSDIGSWGPFAAIAAIIAAIAAIFPFLSGIVKF
His6-SpyCatcher: GFP-StrepTag	MHHHHHHVTTLSGLSGEQGPSGDMTTEEDSATHIKFSKRDEDGRELAGATMELRDSSGKTISTWISDGHVKDFYLYPGKYTFVETAAPDGYEVATPIEFTVNEDGQVTVDGEATEGDAHTSGGGGSSGSKGEELFTGVVPILVELDGDVNGHKFSVRGEGEGDATNGKLTLKFICTTGKLPVPWPTLVTTLTYGVQCFSRYPDHMKQHDFFKSAMPEGYVQERTISFKDDGTYKTRAEVKFEGDTLVNRIELKGIDFKEDGNILGHKLEYNFNSHNVYITADKQKNGIKANFKIRHNVEDGSVQLADHYQQNTPIGDGPVLLPDNHYLSTQSVLSKDPNEKRDHMVLLEFVTAAGITHGMDELYKGSWSHPQFEK

Name	DNA Sequence
PS2 promoter	GAATTCCTGTGAATTAGCCGGTTTAGTACTTTTCAGGGGTGTCTATTCTTACCAGATCGTCAAGTTGTGGGTAGAGTCACCTGAATATTAATTGCACCGCACGGGTGATATATGCTTATTTGCTCAAGTAGTTCGAGGTTAAGTGTATTTTAGGTGAACAAATTTCAGCTTCGGGTAGAAGACTTTCTATGCGCTTCAGAGCTTCTATTAGGAAATCTGACACCACTTGATTAAATAGCCTACCCCCGAATTGGGGGATGGGTCATTTTTTGCTGTGAAGGTAGTTTTGATGCATATGACCTGCGTTTATAAAGAAATGTAAACGTGATCAGATCGATATAAAAGAAACAGTTTGTACTCAGGTTTGAAGCATTTTCTCCGATTCGCCTGGCAAAAATCTCAATTGTCGCTTACAGTTTTTCTCAACGACAGGCTGCTAAGCTGCTAGTTCGGTGGCCTAGTGAGTGGCGTTTACTTGGATAAAAGTAATCCCATGTCGTGATCAGCCATTTTGGGTTGTTTCCATAGCAATCCAAAGGTTTCGTCTTTCGATACCTATTCAAGGAGCCTTCGCCTCT

Table S2. Sequences of interest used in this study. The SpyTag sequence is underlined.

Appendix Table Cryo-EM model and data statistics	*Ex vivo* PS2 (EMDB-51414)(PDB 9GK2)
**Data collection and processing**	CryoARM300, BECM
Magnification	60,000
Voltage (kV)	300
Electron exposure (e–/Å^2)	60
Defocus range (μm)	-1.1 to -1.4
Pixel size (Å)	0.71
Symmetry imposed	C6
Tilts collected	30 and 15
Final particle images (no.)	521.917
Map resolution (Å)	2.51*
FSC threshold	0.143
Map resolution range (Å)	2.5-3.8
**Refinement**	
Initial model used	AlphaFold2
Model resolution (Å)	2.44
FSC threshold	0.143
Model resolution range (Å)	-
**Model composition**	
Non-hydrogen atoms	60516
Protein residues	7776
Ligands	0
**B factors (Å^2)**	
Protein (mean)	53.80
Ligand	NA
**R.m.s. deviations**	
Bond lengths (Å)	0.004
Bond angles (°)	0.764
**Validation**	
MolProbity score	2.28
Clashscore	9.81
Poor rotamers (%)	4.10
**Ramachandran plot**	
Favored (%)	95.63
Allowed (%)	4.01
outliers (%)	0.36

* as from the 3d FSC job

Table S3. Cryo-EM data collection, refinement and validation statistics of the PS2 structure.

SI Methods

Unfolding and refolding of the PS2 S-layer

Recombinant His-PS2AD was purified as previously described. The gel-like fraction obtained after washing (described above) was resuspended overnight in unfolding buffer (8 M urea, 500 mM NaCl, 50 mM Hepes pH 7). The following day, the protein was loaded onto a SpinTrap column (Cytiva) pre-equilibrated with Buffer A, and washed five times with refolding buffer (500 mM NaCl, 50 mM Hepes, pH 7) with or without 10 mM EDTA. After washing, the proteins were eluted with Buffer B, also with or without 10 mM EDTA, and incubated overnight at 20°C before examination via ns-EM.

Negative-stain transmission electron microscopy (TEM)

For visualisation of the PS2 S-layers by negative stain TEM, carbon-coated copper grids with 400-hole mesh (Electron Microscopy Science) were glow discharged (ELMO; Agar Scientific) with a plasma current of 5mA at vacuum for 60 s. Freshly glow-discharged grids were used immediately by applying 4 µl of sample (either purified PS2 or extracted directly from C. glutamicum cells) and allowing binding to the support film for 1 min after which the excess liquid was blotted away with Whatman grade 2 filter paper. The grids were then washed three times using three 15 µl drops of ddH2O followed by blotting of excess liquid. The washed grids were held in 15 µl drops of 2% uranyl acetate three times for, respectively, 10 s, 2 s, and 1min duration, with a blotting step in between each drop. Finally, the uranyl acetate-coated grids were fully blotted. The grids were then imaged using a 120 kV JEOL 1400 microscope equipped with LaB6 filament and TVIPS F416 CCD camera.

Phylogenetic analysis

We assembled a database containing all 2325 Corynebacterium genomes and proteomes present at the GenBank database (Sayers et al., 2022) as of January 2024. We used HMM profile searches to identify protein PS2 in the protein database. First, we used the HMMER package (v3.3.2) (L. S. Johnson et al., 2010) tool jackhmmer to look for homologs of *C. glutamicum* PS2 in all the proteomes using the GenBank sequence AAX43986.1 as query. The hits were aligned with mafft (v7.475) (Katoh et al., 2005) using default parameters. The alignments were manually curated, removing sequences that did not align globally. The hits obtained by jackhmmer might not include sequences that are very divergent from the single sequence query. For this reason, the alignment was used to create an HMM profile using the HMMER package (v3.3.2) tool hmmbuild. This specific and curated HMM profile of PS2 was used for a second and final round of searches against the proteomes using the HMMER tool hmmsearch. The new hits were aligned with linsi, the accurate option of mafft (v7.475), and trimmed using bmge (1.12) (Criscuolo & Gribaldo, 2010). The trimmed alignment was used to reconstruct the phylogeny of PS2. We repeated the search of PS2 against a database containing all *Corynebacteriales* order diversity (Gaday et al., 2022), obtaining no new hits. We inferred a maximum-likelihood tree of PS2 with IQ-TREE (Nguyen et al., 2015), using the posterior mean site frequency (PMSF) and the model LG + C60 + F + G, with

ultrafast bootstrap supports calculated from 10,000 replicates. The guide tree required by the PMSF model was obtained using the LG+G+I+F model and the same trimmed alignment. To compare the genomic contexts of PS2, we retrieved 10 genes upstream and downstream of each PS2 hit, and we annotated the corresponding proteins using EggNOG-mapper (v2.1.12) (Huerta-Cepas et al., 2017) with the default parameters. The genomic context of PS2 in each strain was mapped on the *Corynebacterium* PS2 phylogeny using the online tool iTOL (Letunic & Bork, 2019) and custom scripts. We reconstructed a reference phylogeny of *Corynebacterium*, based on protein RNA polymerase subunit B, using the method described for protein PS2. We also reconstructed a reduced reference phylogeny of *Corynebacterium*, selecting only one strain per species (175 species), and a reduced phylogeny containing only the strains where PS2 was identified (102 taxa).

SI references

1. Ashkenazy H, Abadi S, Martz E, et al. ConSurf 2016: an improved methodology to estimate and visualize evolutionary conservation in macromolecules. *Nucleic Acids Res*. 2016;44(W1):W344-W350. doi:10.1093/nar/gkw408.
2. Crooks GE, Hon G, Chandonia JM, Brenner SE. WebLogo: a sequence logo generator. Genome Res. 2004;14(6):1188-1190. doi:10.1101/gr.849004
3. Peyret JL, Bayan N, Joliff G, Gulik-Krzywicki T, Mathieu L, Schechter E, Leblon G. Characterization of the cspB gene encoding PS2, an ordered surface-layer protein in Corynebacterium glutamicum. Mol Microbiol. 1993 Jul;9(1):97-109. doi: 10.1111/j.1365-2958.1993.tb01672.x.
